# Globally altered epigenetic landscape and delayed osteogenic differentiation in H3.3-G34W-mutant giant cell tumor of bone

**DOI:** 10.1038/s41467-020-18955-y

**Published:** 2020-10-27

**Authors:** Pavlo Lutsik, Annika Baude, Daniela Mancarella, Simin Öz, Alexander Kühn, Reka Toth, Joschka Hey, Umut H. Toprak, Jinyeong Lim, Viet Ha Nguyen, Chao Jiang, Anand Mayakonda, Mark Hartmann, Felix Rosemann, Kersten Breuer, Dominik Vonficht, Florian Grünschläger, Suman Lee, Maren Kirstin Schuhmacher, Denis Kusevic, Anna Jauch, Dieter Weichenhan, Jozef Zustin, Matthias Schlesner, Simon Haas, Joo Hyun Park, Yoon Jung Park, Udo Oppermann, Albert Jeltsch, Florian Haller, Jörg Fellenberg, Anders M. Lindroth, Christoph Plass

**Affiliations:** 1 Division of Cancer Epigenomics, German Cancer Research Center (DKFZ), Im Neuenheimer Feld 280, 69120 Heidelberg, Germany; 2 Faculty of Biosciences, Ruprecht Karl University of Heidelberg, Im Neuenheimer Feld 234, 69120 Heidelberg, Germany; 3 Bioinformatics and Omics Data Analytics, German Cancer Research Center (DKFZ), Im Neuenheimer Feld 280, 69120 Heidelberg, Germany; 4 Graduate School of Cancer Science and Policy, Cancer Biomedical Science, National Cancer Center, Goyang-si, Gyeonggi-do, 10408 Republic of Korea Republic of Korea; 5 Botnar Research Centre, Oxford NIHR BRC, Nuffield Department of Orthopaedics, Rheumatology and Musculoskeletal Sciences, University of Oxford, Oxford, OX3 7LD UK; 6 Section Translational Cancer Epigenomics, Division of Translational Medical Oncology, National Center for Tumor Diseases (NCT) & German Cancer Research Center (DKFZ), Im Neuenheimer Feld 460, 69120 Heidelberg, Germany; 7 Division of Stem Cells and Cancer, German Cancer Research Center (DKFZ), Im Neuenheimer Feld 280, 69120 Heidelberg, Germany; 8 Heidelberg Institute for Stem Cell Technology and Experimental Medicine–HI-STEM gGmbH, Im Neuenheimer Feld 280, 69120 Heidelberg, Germany; 9 Department of Biochemistry, Institute of Biochemistry and Technical Biochemistry, University of Stuttgart, Allmandring 31, 70569 Stuttgart, Germany; 10 Institute of Human Genetics, Ruprecht Karl University of Heidelberg, Im Neuenheimer Feld 366, 69120 Heidelberg, Germany; 11 Institute of Pathology, University Medical Center Hamburg-Eppendorf, Martinistrasse 52, 20251 Hamburg, Germany; 12 Department of Nutritional Science and Food Management, Ewha Womans University, 52 Ewhayeodae-gil, Daehyeon-dong, Seodaemun-gu, Seoul 03760 Republic of Korea; 13 FRIAS-Freiburg Institute of Advanced Studies, Albert Ludwig University of Freiburg, Alberstrasse 19, 79104 Freiburg, Germany; 14 Institute of Pathology, University Hospital Erlangen, Friedrich Alexander University Erlangen-Nürnberg, Krankenstrasse 8, 91054 Erlangen, Germany; 15 Department of Experimental Orthopaedics, Orthopaedic University Hospital Heidelberg, Ruprecht Karl University of Heidelberg, Schlierbacher Landstrasse 200a, 69118 Heidelberg, Germany; 16 German Consortium for Translational Cancer Research (DKTK), Heidelberg, Germany; 17 Present Address: Division of Neuroblastoma Genomics, German Cancer Research Center (DKFZ), Im Neuenheimer Feld 280, 69120 Heidelberg, Germany

**Keywords:** Data mining, Cancer genomics, DNA methylation, Epigenomics, Bone cancer

## Abstract

The neoplastic stromal cells of giant cell tumor of bone (GCTB) carry a mutation in *H3F3A*, leading to a mutant histone variant, H3.3-G34W, as a sole recurrent genetic alteration. We show that in patient-derived stromal cells H3.3-G34W is incorporated into the chromatin and associates with massive epigenetic alterations on the DNA methylation, chromatin accessibility and histone modification level, that can be partially recapitulated in an orthogonal cell line system by the introduction of H3.3-G34W. These epigenetic alterations affect mainly heterochromatic and bivalent regions and provide possible explanations for the genomic instability, as well as the osteolytic phenotype of GCTB. The mutation occurs in differentiating mesenchymal stem cells and associates with an impaired osteogenic differentiation. We propose that the observed epigenetic alterations reflect distinct differentiation stages of H3.3 WT and H3.3 MUT stromal cells and add to H3.3-G34W-associated changes.

## Abstract (web summary)

The histone variant mutation H3.3-G34W occurs in the majority of giant cell tumor of bone (GCTB). By profiling patient-derived GCTB tumor cells, the authors show that this mutation associates with epigenetic alterations in heterochromatic and bivalent regions that contribute to an impaired osteogenic differentiation and the osteolytic phenotype of GCTB.

## Introduction

The discovery of mutated histone genes in aggressive cancers raised a lot of interest in the cancer research community due to their ability to globally alter the epigenomic landscape^1^. A frequently mutated histone is the non-canonical histone variant H3.3^2,3^. In contrast to canonical H3.1 and H3.2, the incorporation of histone variant H3.3 is replication-independent and its turnover occurs throughout the cell cycle^4^. Deposition occurs either via the histone regulator A (HIRA) chaperone complex at sites of gene activation or through alpha-thalassemia/mental retardation X-linked-death domain associated protein (ATRX-DAXX) into heterochromatic regions^5^ and the silent allele of imprinted genes^6^. Mouse embryonic stem cells require H3.3 for correct establishment of H3K27me3 patterns at bivalent promoters of developmentally regulated genes^7,8^. While two human genes, H3 histone family member 3A (*H3F3A*) and 3B (*H3F3B*), encode for an identical H3.3 protein, oncogenic mutations occur gene-specifically in different tumor types. Lysine-27-to-methionine (H3.3-K27M) and glycine-to-arginine or valine substitution (H3.3-G34R/V) in pediatric gliomas^9^, as well as glycine-34-to-tryptophan or leucine (H3.3-G34W/L) substitutions in giant cell tumor of bone (GCTB)^10^ have been described, all due to mutations in *H3F3A*. For *H3F3B*, mutations leading to a lysine-36-to-methionine (H3.3-K36M) substitution were reported in chondroblastomas^10,11^. The molecular consequence of the H3.3-K27M mutation is a global loss of the repressive chromatin mark H3K27me3 through inactivation of the PRC2 complex^12–14^. Similarly, the H3.3-K36M mutation suppresses the deposition of the H3K36me3 mark by interference with histone methyltransferases nuclear receptor binding SET domain protein 2 (NSD2) and SET domain containing 2 (SETD2)^11,15^. Recent findings suggested reduced levels of H3K36me3 and increased levels of H3K27me3 in *cis* in HeLa cells overexpressing H3.3-G34W^16^. However, the detailed effects of this mutant histone variant on the epigenome are yet to be determined and to be analyzed in patients. GCTB, where this mutation was shown as the sole alteration, offers a unique system to study these effects in primary patient material.

GCTB is a rare locally aggressive bone neoplasm that typically affects the meta-epiphyseal regions of long bones in young adults^17^. These tumors consist of three major cell types: stromal cells originating from mesenchymal stem cells (MSC), multinuclear giant cells and mononuclear histiocytic cells^18^. The GCTB stromal cells show incidence of H3.3-G34W in more than 90% of cases and display markers of both MSC and pre-osteoblast cell populations^10,19^. The neoplastic stromal cell population secretes high levels of the receptor activator of NF-κB ligand (RANKL) and reduced levels of its decoy receptor, osteoprotegerin (OPG), thereby attracting and activating surrounding monocytes. Upon activation, the recruited monocytes fuse to form multinucleated giant cells, which resemble osteoclasts and lead to massive bone destruction^17^.

Here we investigate the effects of H3.3-G34W on global epigenomic patterns in patient samples from four different centers. We find epigenetic distortions that contribute to the phenotypes of GCTB, stochastic genomic instability and increased osteolysis. Furthermore, we demonstrate that neoplastic and non-neoplastic GCTB stromal cells represent distinct stages of osteogenic differentiation. Differentiation-related epigenetic differences add to the overall picture of H3.3-G34W-associated global epigenetic alterations, whereas the differentiation delay is potentially driven by the direct effects of H3.3-G34W. Our findings collectively suggest that the single-residue alteration of H3.3 induces epigenomic changes with implications for the development of stromal cells and the tumorigenic process.

## Results

### H3K36me is unaltered in H3.3-G34W-expressing stromal cells

Recent biochemical studies have shown that G34 substitutions in H3.3, including G34W, inhibit the activity of the histone methyltransferase SETD2, which is responsible for H3K36me3, in *cis* (on the same histone tail)^16^. We verified this effect in HEK293T cells stably overexpressing H3.3-G34W or wild-type H3.3 as a control and confirmed in *cis* effects on H3.3-G34W K36 trimethylation levels (Supplementary Fig. 1a). We did not observe any in *trans* effects on endogenous H3 modifications (Supplementary Fig. 1a), as found for other mutant histones such as H3-K36M.

**Fig. 1:**
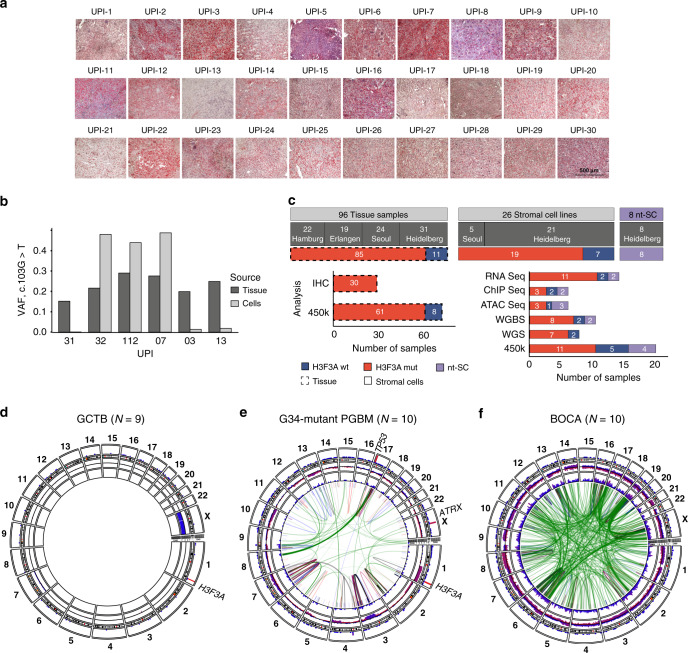
Initial characterization of GCTB patient samples. **a** Immunohistochemical staining of primary GCTB tumor resections with a H3.3-G34W-specific antibody (Active Motif) (red). UPI, unified patient identifier. The scale bar exemplarily shown for UPI-30 indicates 500 µm. **b** Quantification of the mutation at position 103 in the *H3F3A* gene (c.103G>T) leading to the H3.3 G34W substitution in tumor resections (dark gray) and derived stromal cell lines (light gray) using deep targeted resequencing. VAF, variant allele frequency. **c** Overview of GCTB tissues and derived stromal cell lines (*H3F3A* wt in blue, mut in red, non-tumoral stromal cells (nt-SC) in violet) analyzed within this study. IHC, immunohistochemistry; WGS, whole genome sequencing; WGBS, whole genome bisulfite sequencing; 450 K, DNA methylation array. **d**–**f** Circos plot of recurrent structural variants in GCTB (**d**), H3.3-G34R-baring pediatric glioblastoma (PGBM, **e**) and bone cancer (BOCA-UK, **f**), cohorts based on whole genome sequencing data. Green lines represent translocations, blue lines deletions, red lines duplications, and black lines inversions. The variant recurrences are represented by bar plots. The outermost layer represents functional small variants (SNVs and small indels). The middle layer represents copy number variations. The innermost layer represents structural variations. All layers are normalized to the compared cohort size. Osteosarcoma cohort was sub-sampled at random to the size of the other two cohorts. See high-resolution versions in Supplementary Fig. 1f–h.

To specifically test whether these biochemical findings apply to patient samples, we obtained access to GCTB biopsies from four different cohorts (Supplementary Data 1). For the initial characterization of 30 GCTB samples (Supplementary Data 1), we performed immunohistochemical analysis with a H3.3-G34W-specific antibody. Positive staining was observed and validated in 29 of 30 cases (Fig. 1a, b and Supplementary Fig. 1b). The H3.3-G34W-negative case (unified patient identifier, UPI-13) carried a *H3F3A* (c.103_104GG>TT) mutation encoding a H3.3-G34L substitution that has already been described for GCTB^10^ (Fig. 1b, Supplementary Fig. 1c). We established both, neoplastic, H3.3 G34W-expressing (H3.3 MUT) and non-neoplastic, H3.3-G34W negative (H3.3 WT) stromal cell lines from primary tumor tissue (Fig. 1b; Supplementary Data 1). Cell type differences were ruled out by flow cytometric analyses which revealed a high expression of MSC markers and low expression of hematopoietic markers in both H3.3 WT and H3.3 MUT cell lines, suggesting that both are of mesenchymal origin (Supplementary Fig. 1d). In total, we collected 96 tissue samples from 95 different GCTB patients from four different cohorts (Supplementary Data 1) and were able to establish 26 stromal cell lines from 24 different GCTB patients from two cohorts (Fig. 1c). In addition to H3.3 WT cells, we analyzed bone marrow-derived primary MSCs from non-GCTB patients from here on referred to as nontumoral stromal cells (nt-SC) (Supplementary Data 1). We verified the mutational status of the cells using Sanger resequencing, ultra-deep resequencing on the MiSeq platform or whole-genome sequencing (Supplementary Data 1, Fig. 1b, Supplementary Fig. 1c, e). In addition to the common mutation leading to the G34W alteration in H3.3, whole-genome sequencing of seven patient-derived primary H3.3 MUT cell lines (Supplementary Data 1) did not reveal any recurrent genetic alterations (Fig. 1d, Supplementary Fig. 1e, f). In contrast to other malignancies carrying *H3F3A* mutations, as for example pediatric glioblastoma with co-occurring mutations in tumor protein 53 (*TP53)* and *ATRX*^9,20^ (Fig. 1e, Supplementary Fig. 1g), and to other bone tumors, e.g., osteosarcoma (Fig. 1f, Supplementary Fig. 1h, i), GCTB showed an extremely low overall mutation frequency for H3.3 MUT and H3.3 WT cells (Fig. 1f, Supplementary Fig. 1e, f). This places GCTB in a unique position to uncover epigenomic alterations linked to H3.3-G34W.

To understand how H3.3-G34W exerts its function in tumor cells, we analyzed protein fractions by Western Blot and found H3.3-G34W incorporated into chromatin (Supplementary Fig. 2a). To analyze in *cis* effects of H3.3-G34W in patient derived cells, we used a specific and verified antibody to map H3.3-G34W and identify sites of H3.3-G34W enrichment in two independent patient cell lines (Supplementary Data 1). We identified high-confidence genomic regions showing H3.3-G34W enrichment (Supplementary Fig. 2b, c, Supplementary Data 2) and profiled H3K36me3 along with several other histone modifications using chromatin immunoprecipitation (ChIP-mentation). Changes of H3K36me3 or H3K27me3 between H3.3 WT and H3.3 MUT stromal cells at sites of H3.3-G34W enrichment were minute and did not recapitulate the strong loss of H3K36me3 in *cis* observed in HEK293T cells (Supplementary Fig. 2b, c). Nucleosomes enriched by H3.3-G34W-ChIP most probably contain H3.3-G34W, as well as wild-type H3.3 potentially masking in *cis* effects on the H3.3-G34W N-terminal tail. Using Western Blot analysis of whole cell lysates we did not reveal any changes in the total amount of K36 methylated histone H3 indicating that the G34W substitution does not affect methylation of K36 on other histones lacking the mutation, which is in line with the analysis in HEK293T cells in Supplementary Fig. 1a (Supplementary Fig. 2d).

### The epigenome of H3.3 MUT cells is globally altered

**Fig. 2:**
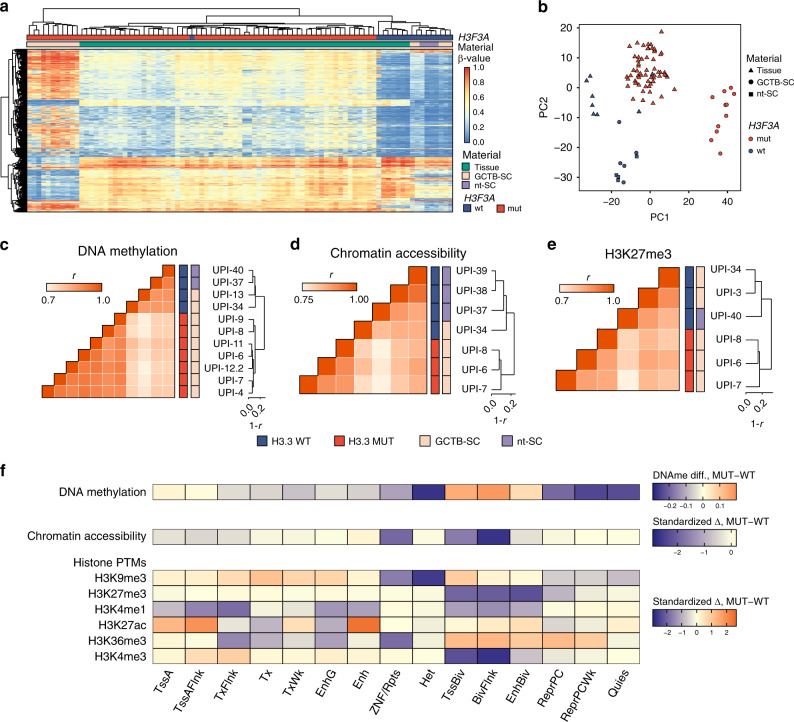
Genome-wide epigenetic distortion in H3.3 MUT stromal cells. **a** HumanMethylation450 profiles of GCTB tumor tissue samples (green), GCTB stromal cells (GCTB-SC in pink, wt in blue, mut in red) and healthy nontumoral stromal cells (nt-SC, violet). Heatmap displays 10,000 CpG sites with highest variance across all samples. Agglomerative, hierarchical clustering of rows (CpGs) and columns (samples) was performed with average-linkage based on Euclidean distance metric. **b** Principal component analysis of HumanMethylation450 array-based DNA methylation profiles of GCTB tumor tissue (triangles), GCTB stromal cells (GCTB-SC, circles) and nontumoral stromal cells (nt-SC, rectangles). Samples with wildtype (wt) and mutant (mut) *H3F3A* status are shown in blue and red, respectively. Each data point represents one patient. **c**–**e** Hierarchical clustering with correlation distance of DNA methylation (**c**), chromatin accessibility (**d**) and H3K27me3 (**e**) profiles in nt-SC (violet) and stromal cells (pink). H3.3 mutational groups in blue (H3.3 WT) and red (H3.3 MUT). Heatmaps represent pairwise Pearson correlation coefficients (*r*) between respective modification profiles of two cell lines. Dendrograms were obtained with agglomerative hierarchical clustering with 1-*r* distance and average linkage. Heatmap color codes represent the H3.3 mutational status (inner), and the cell type (outer). UPI, Unified patient identifier. **f** Stratification of epigenetic differences using MSC-specific chromatin states as defined by ChromHMM. For DNA methylation difference between average methylation levels of all CpGs falling into corresponding states in H3.3 WT and H3.3 MUT cells is presented. For chromatin accessibility and histone post-translational modifications (PTMs), the standardized difference (delta over root-mean-square of deltas in each row) between average normalized read counts over all regions of a state is shown (values are only row-wise comparable). Mnemonics for the ChromHMM states are defined by the Roadmap project (TssA, active TSS; TssFlnk, active TSS flanking regions; Tx, transcribed regions; TxFlnk, transcription franking regions; TxWk, weakly transcribed regions; Enh, enhancers; EnhG, genic enhancers, ZNF/Rpts; zinc finger genes and repeats; TssBiv, bivalent TSS; BivFlnk, flanking bivalent regions; EnhBiv, bivalent enhancer; ReprPC, Polycomb repressed; ReprPCWk, weak Polycomb repressed; Quies, quiescent). All analyzed stromal cell lines with their UPIs are listed in Supplementary Data 1.

As the effects of H3.3-G34W on the global level of H3.3K36me3 were insignificant, we tested whether H3.3-G34W might exert its tumorigenic effects through other epigenetic mechanisms. We analyzed several H3.3 MUT and H3.3 WT cells, as well as a large number of biopsies of the four GCTB cohorts (Supplementary Data 1), using HumanMethylation450 arrays (89 samples in total) and observed full-range DNA methylation changes of a large number of CpG sites (Fig. 2a). Dimensionality reduction and clustering analysis of the methylation data showed the most pronounced changes between primary patient-derived H3.3 MUT and H3.3 WT cell lines as compared to GCTB biopsies. Most of the primary tumor samples had an intermediate methylome identifying them as mixtures of normal and tumor cells (Fig. 2a, b), as also confirmed by deep resequencing of the *H3F3A* gene locus of tissue-derived DNA showing a lower variant allele frequency than the theoretically expected 0.5 for a heterozygous mutation (Fig. 1b). This observation prompted us to restrict all our subsequent analyses to H3.3 MUT and H3.3 WT cells in order to obtain a clean view of H3.3-G34W-associated epigenetic alterations in pure cell populations. To further a detailed analysis of the epigenome, we profiled DNA methylation at single CpG resolution using whole genome bisulfite sequencing (WGBS), analyzed chromatin accessibility with the assay for transposase-accessible chromatin using sequencing (ATAC-seq) and analyzed global distribution of several histone marks (H3K4me1, H3K4me3, H3K9me3, H3K27me3, H3K27ac, H3K36me3) to investigate their potential redistribution. Since the initial methylation analysis showed high homogeneity within the mutant and wild-type groups and culturing primary tumor cells did not yield sufficient material for a comprehensive omics profiling of each line, we analyzed H3.3 WT and H3.3 MUT cell lines from several patients to exclude patient-dependent alterations (Supplementary Data 1). Global hierarchical clustering proved the H3.3-G34W substitution to be the major determinant of variability in DNA methylation, chromatin accessibility and posttranslational histone modifications (H3K27me3, H3K27ac, H3K4me1) with H3.3 MUT and H3.3 WT groups forming separate clusters (Fig. 2c–e, Supplementary Fig. 2e). In contrast, other histone modifications (H3K36me3, H3K4me3) did not result in H3.3 MUT-separating and H3.3 WT-separating clusters (Supplementary Fig. 2e). Since nt-SCs clustered with H3.3 WT cells, we combined all H3.3 wild type cells into a single control group (H3.3 WT) for further analysis.

In order to identify loci with more pronounced epigenetic changes, we used a genome-wide approach to stratify the observed differences from all epigenetic layers using the ENCODE functional annotation of the MSC epigenome^21^. We observed profound differences in several groups of chromatin states including heterochromatic and Polycomb-repressed states, as well as bivalent domains (Fig. 2f**)**. Heterochromatic regions were noticeably hypomethylated while bivalent domains accumulated a whole range of alterations, including gain of DNA methylation, loss of chromatin accessibility, as well as loss of several histone modifications (H3K27me3, H3K4me3 etc). Collectively, we conclude that the epigenome of H3.3 MUT cells shows significant and reproducible differences to that of H3.3 WT stromal cells.

### Hypomethylation in megabase domains and genomic instability

**Fig. 3:**
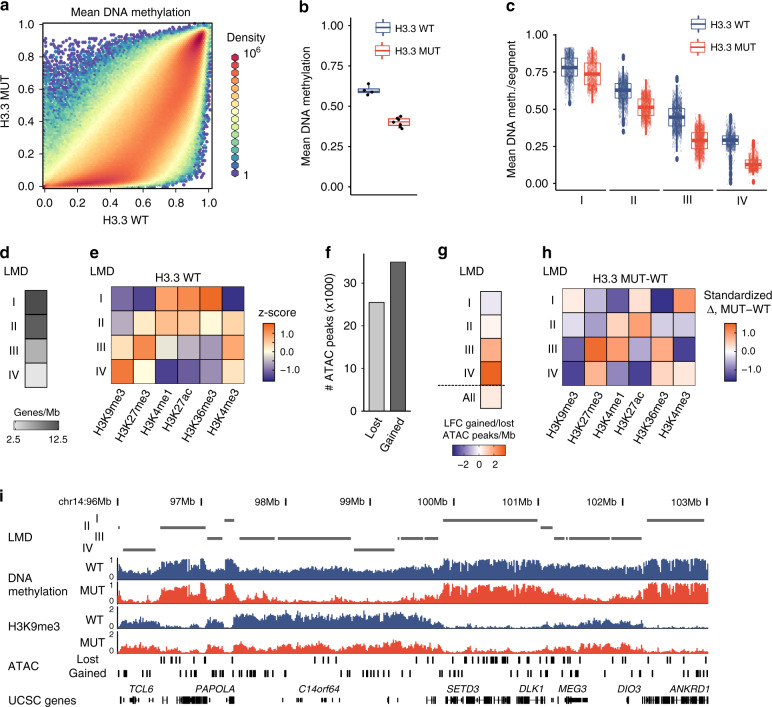
Heterochromatin defects in H3.3 MUT stromal cells. **a** Binned scatterplot of DNA methylation profiles from WGBS of H3.3 WT cells (*x*-axis) versus H3.3 MUT cells (*y*-axis). Hexagon color represents binned density gradient of 1 (blue), 1000 (yellow), and 10^6^ (red). **b** Genome-wide mean level of DNA methylation in H3.3 WT (blue) and H3.3 MUT (red) cells. Each data point represents one patient, H3.3 WT *n* = 4 and H3.3 MUT *n* = 7 biologically independent cell lines. The difference is statistically significant (*p* = 8.3 10^−3^, unpaired two-tailed *t*-test). **c** Methylation level distribution at large methylation domains (LMDs) in H3.3 WT (blue) and H3.3 MUT (red) cells. Each point represents a single LMD segment. **d** Mean gene density of LMDs. MB, mega bases. **e** Normalized levels of histone modifications at LMDs in H3.3 WT cells. Heatmap represents *z*-scores of average signals across all segments within the LMDs calculated using overall mean level and standard deviation across all LMD segments. **f** Absolute numbers of ATAC-seq peaks with differential chromatin accessibility between H3.3 WT cells and H3.3 MUT cells. Lost (light gray) and gained (dark gray) refer to peaks showing a decrease (disappearance) or increase (emergence) in H3.3 MUT cells, respectively. **g** Stratification of differential chromatin accessibility between H3.3 WT and H3.3 MUT cells by overlap with LMDs. Labels as in **f**; all, distribution of ATAC peaks across all LMDs. **h** Changes of histone modifications between H3.3 WT and H3.3 MUT stromal cells at LMDs. Heatmap represents standardized difference (delta over root-mean-square of all deltas in each column) between average normalized read counts over all regions of a state is shown (values are only column-wise comparable). **i** Genomic browser view of WGBS, H3K9me3 ChIP-seq and ATAC-seq in a representative 7Mbp region on chromosome 14 (96,000,000–103,000,000). Tracks represent average profiles for H3.3 WT (blue) and H3.3 MUT (red) cells. Bottom panel indicates gene locations based on the UCSC default gene track. Differential ATAC-seq labels as in **f**. All analyzed stromal cell lines with their UPIs are listed in Supplementary Data 1. In all boxplots th bars represents the means, boxes the IQRs, upper and lower whiskers extend from the smallest to the largest value within 1.5 IQR from the lower and upper edges of the box, respectively.

We first followed up changes associated with loss of DNA methylation. In contrast to the homogeneity of DNA methylation profiles observed within each of the two groups (Supplementary Fig. 3a), DNA methylation was non-uniformly altered between H3.3 WT and H3.3 MUT cells across the entire genome (Fig. 3a). The majority of significantly changed loci showed profound reduction of DNA methylation in H3.3 MUT cells, while a minority was hyper-methylated (Fig. 3a, Supplementary Fig. 3b). Overall, H3.3 MUT cells exhibited a 20% genome-wide reduction of DNA methylation (Fig. 3b, Supplementary Fig. 3c). To systematically characterize the abundant DNA methylation changes, we segmented the genome into large methylation domains (LMD I to IV) with sizes >20 kb (Supplementary Fig. 3d, Supplementary Data 2) using a combination of breakpoint analysis and clustering (see Methods for details). The majority of regions (predominantly LMDs III and IV) matched the criteria for partially methylated domains i.e., megabase-scale domains of predominantly repressive chromatin with low gene density^22^. Each LMD had a distinct level of DNA methylation, a discrete pattern of histone marks and gene density, suggesting different functional roles (Fig. 3c–e). For instance, LMD III showed enrichment of H3K27me3, while LMD IV was associated with H3K9me3, suggesting an association with facultative and constitutive heterochromatin (Fig. 3e). Furthermore, LMD III domains were often detected as flanking to LMD IV. LMDs I to IV showed decreasing average methylation levels starting from over 0.75 down to less than 0.25 (Fig. 3c). Lowly methylated LMDs III and IV which could be characterized as heterochromatic (Fig. 3e) showed the most pronounced demethylation comparing H3.3 MUT cells to H3.3 WT cells. We concluded that global DNA methylation alterations in GCTB are non-uniformly distributed along the genome, and that the most pronounced global changes take place in large-scale domains associated with facultative and constitutive heterochromatin, roughly corresponding to LMDs III and IV.

We next sought to associate hypomethylation with other epigenetic alterations. Consistent with the global loss of DNA methylation, we observed increased chromatin accessibility in the H3.3 MUT cells with approximately 1.5-times more gained than lost differential ATAC-seq peaks (Fig. 3f, Supplementary Data 3). ATAC peaks gained in H3.3 MUT cells were overrepresented in the LMDs III and IV (Fig. 3g) indicating a more open state in respective genomic segments. These LMDs also showed strong reduction in the heterochromatic histone mark H3K9me3 (Fig. 3h). Examples of changes in different LMDs can be seen in Fig. 3i. The ATAC peaks gained in H3.3 MUT showed a significant overlap with repetitive elements such as long intersperse nuclear elements (LINE), short interspersed nuclear elements (SINE) and long terminal repeat (LTR) elements which are normally silenced by DNA methylation (Supplementary Fig. 3e, Supplementary Data 3). Looking at other known repetitive regions, such as centromeres and telomers, we found them to be affected by the genome-wide hypomethylation (Supplementary Fig. 3f). Multicolor fluorescence in situ hybridization **(**m-FISH) analysis found H3.3 WT cells to have a normal karyotype, while H3.3 MUT cells in contrast displayed different, non-recurrent centromeric fusions which could be a potential consequence of the heterochromatin defects described above (Supplementary Fig. 3g). We conclude that H3.3-G34W associates with heterochromatic defects that potentially contribute to a genomic instability previously described as characteristic for GCTB^23^.

### Isogenic cells recapitulate H3.3 MUT DNA hypomethylation

**Fig. 4:**
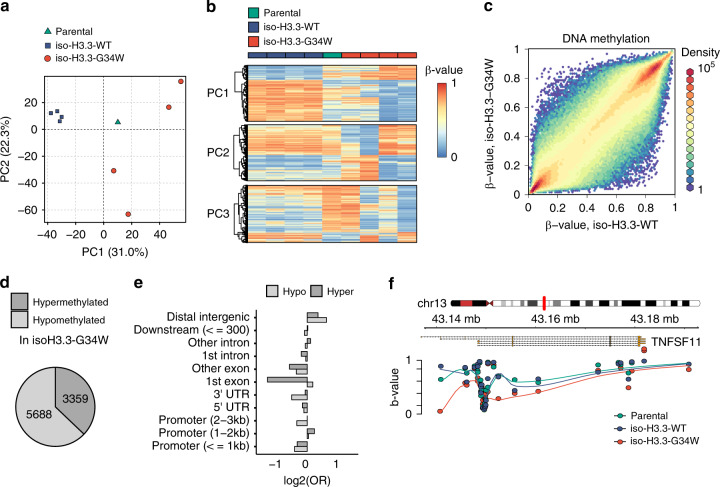
Recapitulation of DNA methylation alterations in an isogenic system. **a** Principal component (PC) analysis of HumanMethylationEPIC profiles of parental (green triangle), iso-H3.3-WT (blue rectangles) and iso-H3.3-G34W (red circle) HeLa cells. Each data point represents an independent clone. **b** Methylation levels of top 2000 probes associated with each of the first three principal components in one parental (green), four iso-H3.3-WT (blue) and four iso-H3.3-G34W cells (red). **c** Scatter plot of average individual CpG probe methylation of four iso-H3.3-WT (*x*-axis) vs. four iso-H3.3-G34W (*y*-axis) clones. Color dots indicate incremental delta means of methylation, with orange representing >0.5 delta-mean. **d** Fractions of HumanMethylationEPIC probes differentially methylated between iso-H3.3-G34W and iso-H3.3-WT HeLa cells, by direction of DNA methylation change (light gray–hypomethylation, dark gray–hypermethylation). **e** Enrichment (positive values) or depletion (negative values) of major gene model features among differentially methylated probes. OR, odds ratio. **f** Genome browser snapshot displaying DNA methylation levels of CpGs in the vicinity of the *TNFSF11* locus in parental (green), iso-H3.3-WT (blue) and iso-H3.3-G34W cells (red). Points represent methylation values of individual CpGs and the lines depict LOESS curves with degree 1 and span 0.5.

**Fig. 5:**
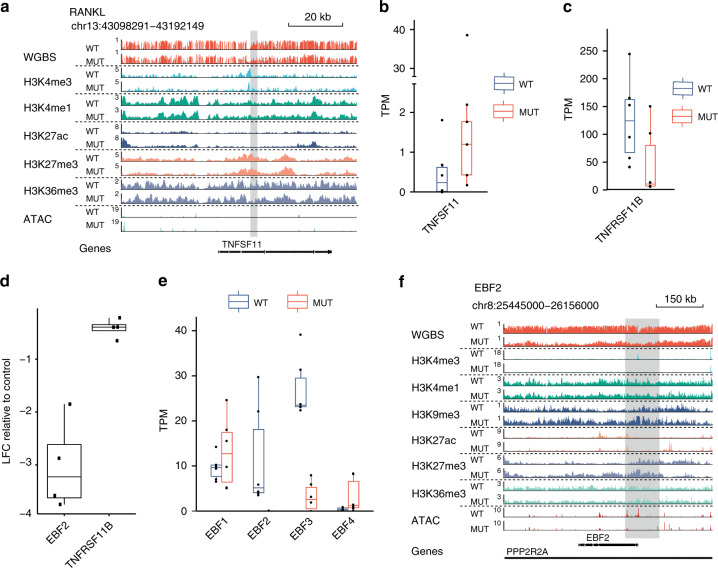
The epigenetic determinants of the osteolytic phenotype in GCTB. **a** Genome browser view of the gene *TNFSF11* encoding for RANKL. Each lane (dash-separated) represents normalized averaged signals of DNA methylation (WGBS), the level of H3K4me3, H3K4me, H3K9me3, H3K27ac, H3K27me3, H3K36me3, and chromatin accessibility (ATAC) in several replicates of H3.3 WT (lane-wise top) or H3.3 MUT (lane-wise bottom) cells. **b, c** Expression levels of RANKL (*TNFSF11*) and OPG (*TNFRSF11B*) in H3.3 WT (blue) H3.3 MUT (red) cells as analyzed by RNA-seq. TPM, transcripts per million. Differences were borderline significant for both genes with *p* = 0.092 (*TNFSF11*) and *p* = 0.062 (*TNFRSF11B*), Wilcoxon rank sum test with *n* = 6 independent patient cell lines in each group. **d** Expression analysis of *EBF2* and OPG (*TNFRSF11B*) by qRT-PCR 48 h after siRNA-mediated knockdown of *EBF2* in H3.3 WT cells (UPI-13). Log fold change (LFC) relative to expression in cells transfected with control siRNAs. *n* = 4 independent experiments are shown. Results showed significance in a one-sample *t*-test with p-values 0.006 and 0.018. **e** Expression levels of all members of the EBF family in H3.3 WT (blue) H3.3 MUT (red) cells as analyzed by RNA-seq. TPM, transcripts per million. Differences were significant for *EBF2* (*p* = 4.8 × 10^−3^) and *EBF3* (*p* = 2.1 × 10^−3^), Wilcoxon rank sum test with *n* = 6 independent patient cell lines in each group. **f** Genomic browser view of the *EBF2* locus. Legend as in **a**. All analyzed stromal cells with their UPIs are listed in Supplementary Data 1. In all boxplots bar represents the mean, box the IQR, upper and lower whiskers extend from the smallest to the largest value within 1.5 IQR from the lower and upper edges of the box, respectively.

As H3.3 MUT stromal cells showed the strongest epigenetic differences to H3.3 WT stromal cells on the DNA methylation level, we expected DNA methylation to play a major role in stromal cell transformation leading to GCTB. To verify that the observed epigenetic changes were dependent on H3.3-G34W expression, we aimed to recapitulate the findings in an unrelated cell line system. To this end, we introduced the H3.3-G34W encoding mutation into HeLa cells by targeting the endogenous *H3F3A* locus as earlier described (Supplementary Fig. 4)^24^. Individual iso-H3.3-WT and iso-H3.3-G34W clones were isolated, of which four iso-H3.3-WT and four iso-H3.3-G34W monoclonal lines and one parental HeLa cell line were subjected to HumanMethylationEPIC DNA methylation analysis (Fig. 4a). The iso-H3.3-G34W samples showed similar alterations as those found when comparing H3.3 MUT and H3.3 WT stromal cells of GCTB (Fig. 4a–e). Principal component analysis of the most variable methylation probes clustered iso-H3.3-WT clones together while iso-H3.3-G34W clones dispersed off, indicating changes in DNA methylation in iso-H3.3-G34W but not WT isogenic cell lines (Fig. 4a). Similar to primary GCTB cells, the largest principal component (PC1, 31% of variance explained) captured widespread hypomethylation in iso-H3.3-G34W accompanied by focal hypermethylation events (Fig. 4a). Clustering analysis further enforced the distinct difference between iso-H3.3-WT and G34W clones (Fig. 4b). A scatter plot of all probes indicated that iso-H3.3-G34W cells showed predominantly hypomethylation as also shown for H3.3 MUT cells from GCTB (Fig. 4c). We found 9047 differentially methylated probes (∆ β-value>0.2 and FDR < 0.05) of which 5688 (63%) were hypomethylated in iso-H3.3-G34W cells (Fig. 4d). These data recapitulate the bias towards hypomethylation found in H3.3 MUT stromal cells in comparison to H3.3 WT stromal cells (compare Fig. 3a, b), and confirm that the changes in DNA methylation are associated with H3.3-G34W. CpG sites that lost methylation were more frequently localized in intergenic regions and promoter-proximal exons, while hypermethylated ones were in addition moderately enriched at promoters and depleted at exons (Fig. 4e). This was coherent with widespread hypomethylation of intergenic regions (LMDs III and IV in Fig. 3c, d, e, h) and overrepresentation of promoters among hypermethylated regions (Supplementary Fig. 3b) observed in H3.3 MUT cells. A specific example for a hypomethylated region in iso-H3.3-G34W cells is the *RANKL* locus showing hypomethylation at exon 3, a potential alternative promoter element (Fig. 4f). This is in line with the methylation differences observed between H3.3 WT and H3.3 MUT stromal cells at the same locus (Fig. 5a). RANKL signaling has been extensively studied in GCTB^25^. The expression of *RANKL*, a master regulator of osteoclast differentiation^26^, has been shown to be upregulated in GCTB stromal cells causing an osteolytic phenotype^25^. We verified the increased expression of *RANKL* (*TNFSF11)* in H3.3 MUT cells (Fig. 5b, Supplementary Fig. 5a) and additionally observed decreased expression and secretion of its decoy receptor Osteoprotegerin (*OPG*, *TNFRSF11B*) (Fig. 5c, Supplementary Fig. 5a, b). Similar expression patterns were already described by us earlier^24^. The *OPG* locus showed decreased levels of the active histone marks H3K4me3 and H3K27ac potentially indicating a missing activation by a transcription factor (Supplementary Fig. 5c). One known transcription factor of *OPG* is the early B-cell factor 2 (EBF2)^27,28^ which is a bivalent gene (Fig. 5f) and belongs to the key regulators of osteogenic differentiation in mice^29^. *OPG* became downregulated after siRNA mediated *EBF2* knockdown, confirming a role of EBF2 in *OPG* expression in stromal cells (Fig. 5d, Supplementary Fig. 5d). The EBF family is a conserved group of four transcription factors. Our RNA-seq analysis found *EBF2* and *EBF3* to be differentially expressed between H3.3 WT and H3.3 MUT stromal cells (Fig. 5e, Supplementary Fig. 5e). *EBF3*, previously reported as a tumor suppressor in glioblastoma^30,31^, showed reduced expression in H3.3 MUT cells whereas *EBF2* expression was almost completely lost. We found the *EBF2* locus to be hypomethylated with a focal hypermethylation around the promoter region (Fig. 5f). Increased H3K9me3 and H3K27me3 levels and decreased levels of H3K27ac supported a repressed state of *EBF2*. Lost ATAC signals in H3.3 MUT cells indicated differentially closed chromatin. These findings link the H3.3 G34W-associated epigenetic dysregulation of *EBF2* expression to the osteolytic phenotype of GCTB.

Taken together, we showed that the stable introduction of H3.3-G34W into a GCTB unrelated cell line recapitulates the DNA hypomethylation trend seen in GCTB stromal cells. Furthermore, we could detect epigenetic alterations that directly and indirectly affected the expression of key regulators of bone metabolism shedding novel light on the emergence of the osteolytic phenotype in GCTB.

### Bivalent promoters and delayed differentiation in H3.3 MUT

**Fig. 6:**
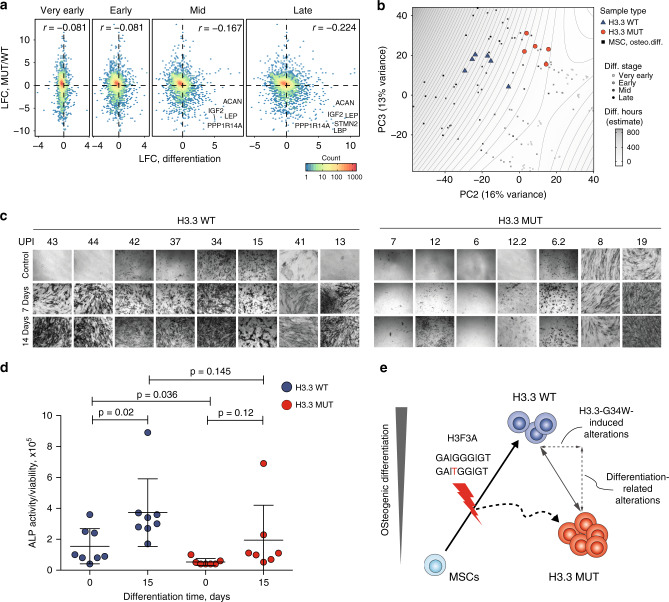
Delay of osteogenic differentiation in H3.3 MUT stromal cells. **a** Correlation between differentiation-ssociated and H3.3-G34W-associated gene expression changes. For the differentiation log-fold changes (LFC) were calculated between average of profiles from each differentiation timepoint group (very early: 0.5 to 2 h; early: 4 to 554 16 h, mid: 24 to 168 h; and late: 336 to 504 h) and the profiles of mesenchymal stem cells (MSCs) sampled prior to the start of the experiment. Each hexagon reflects the density of the underlying data points. Genes with most pronounced changes in both data sets are marked. **b** Principal component analysis of RNA-Seq data from osteogenic differentiation of MSC (greyscale rectangles) and expression profiles of GCTB samples, H3.3 WT (blue triangles) and H3.3 MUT (red circles), only PC2 and PC3 are shown, while PC1 capturing batch effects between two experiments is not shown. The sampled differentiation timepoints were grouped as in **a**. For H3.3 WT and H3.3 MUT each data point represents one patient. The isochores and background shading give a surface fit-based estimate of differentiation time for each point of the (PC2, PC3) plane. **c** Alkaline phosphatase staining of nt-SC, H3.3 WT, and H3.3 MUT cells during osteogenic differentiation. **d** Quantification of alkaline phosphatase (ALP) activity relative to the viability as a surrogate for cell count over the time course of 15 days of in vitro osteogenic differentiation. Each data point represents one patient. Middle bars represent means, error bars show standard deviations within each group. H3.3 WT in blue, *n* = 8 and H3.3 MUT in red, *n* = 7 biologically independent cell lines from different patients. *P*-values are from unpaired (for within-group comparisons) and paired (for between-group comparisons) two-tailed *t*-tests. **e**. Model for GCTB tumorigenesis: The *H3F3A* mutation encoding H3.3-G34W occurs in osteoblastic precursor cells and leads to alterations in osteogenic differentiation. Epigenetic differences described between H3.3 WT and H3.3 MUT stromal cells are the result of measuring cells at different differentiation stages and direct effects of the mutated histone.

Besides dominant hypomethylation in heterochromatic regions, we additionally observed profound epigenetic changes at bivalent domains (Fig. 2f), which are well known sites of H3.3 deposition^5,32^. Changes included gain of DNA methylation which coincide with decreased accessibility, decrease of H3K27me3 and increased levels of H3K36me3 (Fig. 2f). Most promoter-associated ATAC peaks that were lost in H3.3 MUT cells overlapped with bivalent promoters (Supplementary Fig. 6a). As a consequence of epigenetic disturbance, bivalent genes comprised a significant portion of differentially expressed genes both upregulated and downregulated in H3.3 MUT cells (Supplementary Fig. 6b). It was previously reported that comparable epigenetic and transcriptional perturbations at bivalent genes occur in H3.3-deficient mouse ESCs^7^. Genes with decreased expression in H3.3 MUT were significant enriched in Polycomb-target genes, including several developmental transcription factors (Supplementary Fig. 6c, Supplementary Data 4). This was also supported by genomic overlap enrichment analysis of lost ATAC peaks showing strong enrichment at regions where binding of polycomb repressive complex 2 (PRC2) components enhancer of zeste 2 (EZH2) and suppressor of zeste 12 (SUZ12) was found in numerous cell lines (Supplementary Fig. 6d, Supplementary Data 3). Gene ontology (GO) analysis of closed regions revealed many categories related to differentiation (Supplementary Fig. 6e). Impaired differentiation was already suggested for tumor entities harboring mutations in histones including GCTB^15,33^. To investigate whether H3.3 WT and H3.3 MUT cells differ in their osteogenic differentiation state, we performed a comparison across all genes covered by RNA-seq with those obtained from an in vitro differentiation of MSCs. The expression changes during osteogenic differentiation largely correlated inversely with the expression changes observed between H3.3 WT and H3.3 MUT cells, with anticorrelation increasing from very early to late differentiation stages (Fig. 6a). Accordingly, an overlap of the differentially expressed genes from both experiments showed that the majority of matches were upregulated during differentiation and downregulated in H3.3 MUT cells (Supplementary Fig. 6f). Examples included insulin growth factor 2 (*IGF2*) and leptin (*LEP*) previously implicated in osteogenic differentiation^34,35^ (Fig. 6a). A global principal component analysis confirmed a less differentiated state of H3.3 MUT cells resembling MSC in a very early stage of differentiation and H3.3 WT cells showing an expression profile more similar to MSC in early to middle stage of osteogenic differentiation (Fig. 6b). To confirm differences in the osteogenic differentiation state, we stained H3.3 WT and H3.3 MUT stromal cells for the activity of the osteogenic marker alkaline phosphatase (ALP). Most of the H3.3 WT cells exhibited ALP activity whereas only some H3.3 MUT cells showed ALP activity (Fig. 6c). Quantification of the ALP activity relative to viability as a surrogate for cell count confirmed reduced ALP activity for H3.3 MUT stromal cells (Fig. 6d). Impaired differentiation of GCTB stromal cells was suggested earlier based on the analysis of histological markers^36^ and transcriptomic profiling^37^. We performed an osteogenic differentiation of H3.3 MUT and H3.3 WT cells from several patients in vitro to analyze the potential of H3.3 MUT and H3.3 WT cells to differentiate. While both H3.3 WT and H3.3 MUT stromal cells showed an increase of ALP activity indicating the potential for osteogenic differentiation (Fig. 6c, d), we noticed that H3.3 MUT stromal cells lagged behind and did not achieve the level of ALP activity reached by H3.3 WT stromal cells (Fig. 6c, d). We concluded that H3.3-G34W associates with changes in bivalent regions and that their deregulation potentially has an effect on osteogenic differentiation. H3.3 WT and H3.3 MUT stromal cells therefore differ in their state of osteogenic differentiation in GCTB patients and this difference contributes to the herein found epigenetic alterations (Fig. 6e).

## Discussion

Mutant epigenetic regulator proteins, including histones, have been described in multiple cancer types^2^. Despite the fact that oncogenic mutations in histones influence the epigenetic landscape, deep insight into the mechanistic ramifications to cancer initiation is still incomplete. Several studies investigated the mechanism of how the G34W substitution in histone variant H3.3 affects the epigenetic machinery responsible for its post-translational modifications. To this end, reduced H3K36 methylation and increased H3K27me3 in *cis* were described in model systems and verified by us in this work^16,38^. However, whether these effects can be found in patients and whether epigenetic changes are involved in tumorigenesis of GCTB was not known so far. In this paper, we therefore analyzed GCTB tissue, as well as patient-derived primary stromal cells from four different centers to investigate H3.3-G34W-associated epigenetic changes and their contribution to the GCTB neoplasia. The high incidence of H3.3-G34W in GCTB in a largely unaltered genomic context leaves GCTB as a suitable system to study histone mutation-driven tumorigenesis.

We found that, despite that H3.3-G34W is incorporated into chromatin, its presence did not lead to changes in the global amount of H3K36me3 as shown for other substitutions affecting H3.3 lysine 27 and lysine 36^11,13,39^. This ruled out a possible in *trans* effects of H3.3-G34W on histone posttranslational modifications. Using ChIP sequencing, we identified high confidence H3.3-G34W enrichment sites in patient cell lines and analyzed common histone marks at these regions. No pronounced differences were found that could recapitulate the in *cis* effects on H3.3 lysine methylation observed in HEK293T cells. The absence of clear-cut in *cis* effects upon chromatin modifications could be explained by an inability to distinguish wild-type and mutant H3.3 in GCTB samples by western blot analysis. In ChIP analysis, nucleosomes of mutant cells could include both, wild-type, as well as mutated H3.3 obliterating possible in *cis* effects. Moreover, antibodies against histone modifications cannot distinguish between H3 variants and the overall small fraction of the mutant H3.3 (25% of overall H3.3) in the total H3 pool makes analysis of H3.3 modifications challenging.

We show that H3.3-G34W, the sole recurrent alteration of GCTB, associates with large-scale differences in multiple epigenetic marks. H3.3 MUT stromal cells show heterochromatic defects, specifically reduced genome-wide levels of DNA methylation and gained accessibility at heterochromatic regions which is in line with previous reports on pediatric glioblastomas with the G34R substitution in H3.3^39^. These changes could potentially contribute to the genomic instability described for GCTB^23^. We furthermore observed localized changes at bivalent regions, some of the same sites previously described as targets of H3.3 deposition^5,7^. DNA methylation changes could be recapitulated using an isogenic system verifying effects on the methylome to be H3.3-G34W-specific. Moreover, we show H3.3-G34W-associated methylome changes to directly or indirectly affect the expression of the main players of bone metabolism, RANKL and OPG. These results connect the H3.3-G34W-associated epigenetic dysregulation to the osteolytic phenotype, a hallmark of GCTB.

While the epigenomic differences between H3.3 WT and H3.3 MUT stromal cells can be partially ascribed to direct effects of H3.3-G34W, other alterations observed can be explained by the fact that H3.3 MUT and H3.3 WT stromal cells represent distinct differentiation stages. Assuming epigenetic reprogramming during differentiation as demonstrated in recent studies^40,41^, this could also contribute to the epigenetic differences seen between H3.3 WT and H3.3 MUT cells. While expression profiles from H3.3 MUT cells resemble precursor states of osteoblasts, the H3.3 WT cells resemble more mature osteoblasts, suggesting impaired or delayed differentiation of H3.3 MUT cells as already suggested by^42^. A weak differentiation signature is in line with previous reports suggesting that neoplastic GCTB stromal cells are expressing markers of an early osteoblastic differentiation^43^. Genes such as *IGF2*^34^, *LEP*, and stathmin-like 2 (*STMN2*), found to be downregulated in H3.3 MUT stromal cells, were described to play a role in mesenchymal stem cell differentiation and osteoclastogenesis in mice, in promoting bone differentiation^35^ and as a marker for osteogenesis^44^, respectively. Consistently, H3.3 was found to be closely associated with differentiation processes^7,45,46^. The impairment of differentiation of H3.3 MUT stromal cells could be mediated by H3.3-G34W-associated downregulation or prevention of induction of genes upregulated during osteogenic differentiation through a yet unknown mechanism. Epigenetic repression of bivalent genes and dysregulation of PRC2 targets described in this study could potentially contribute to the observed phenotype. H3.3 MUT stromal cells are still able to undergo osteogenic differentiation, as it was also found in studies not separating H3.3-mutant neoplastic from H3.3-wild-type stromal cells of GCTB tissue^47,48^.

Direct effects of H3.3-G34W upon the epigenome could be confirmed in isogenic HeLa cells. The effects on DNA methylation were less pronounced in HeLa cells compared to the alterations observed comparing H3.3 WT and H3.3 MUT stromal cells, potentially reflecting the relatively small passage number after the introduction of the *H3F3A* mutation (approximately 20 passages) in the HeLa cells compared to primary cells. Another explanation may be that DNA methylation differences between H3.3 WT and MUT stromal cells largely reflect differentiation-associated epigenomic alterations shown to occur during osteogenesis^41^ as discussed above. A detailed analysis of H3.3-G34W-induced effects on the epigenome and their connection to impeded osteogenic differentiation will require systems capable of reproducing the differentiation context in vitro or in vivo, such as MSC-derived immortalized cell lines or animal models. Alternatively, detailed transcriptomic and epigenomic maps of the osteogenic differentiation can be instrumental for delineating H3.3-G34W-induced and differentiation-related alterations. Furthermore, meticulous computational approaches, e.g.,^49^, may help disentangle differentiation-related from cancer-specific epigenetic alterations.

Taken together, our results suggest that the molecular mechanism behind H3.3-G34W-induced epigenomic alterations and neoplastic transformation in GCTB is different to the action of other mutant histone variants such as H3.3-K27M and H3.3-K36M, where the mutated amino acid is a direct target for histone modification^11,13,15^. This suggests that the H3.3-G34W substitution alters the N-terminal interactome as previously described^24^, recruiting novel binders or disrupting interactions with binders. The described DNA methylation changes can be indicative of H3.3-G34W affecting either the binding or the activity of enzymes writing (DNA methyltransferases, DNMTs) or erasing (ten eleven translocators, TETs) this modification. On the structural side it is known that DNMT3a/b recognize H3K36me3 with their PWWP-domains^50,51^. The G34W substitution might interfere with binding of DNTM3a/b PWWP preventing proper establishment of DNA methylation patterns. The thereby induced remodeling of the epigenome associated with H3.3-G34W, in particular changes at heterochromatic and bivalent regions, could contribute to an impaired osteogenic differentiation and the phenotypes of GCTB: stochastic chromosomal rearrangements and increased RANKL signaling. Precise sequences of molecular events leading to these phenotypes will be a matter of further studies.

## Methods

### Patient samples

Biopsies and derived primary cell lines were obtained from patients in the Orthopedic University Hospital, Heidelberg (UHOK), the Korean Cancer Center (KCC), the University Clinic of Leipzig (UKL) and the University Medical Center Hamburg-Eppendorf (UKHE). The complete list of samples used in the study is available in Supplementary Data 1. The use of patient samples and the experiments performed in this study was approved by and in accordance with guidelines and regulations by the Ethics Committees of the University of Heidelberg, University Clinic of Leipzig, University Medical Center Hamburg-Eppendorf, and the National Cancer Center of Korea (IRB NCC2015-0070). Informed consent has been obtained from all patients. All H3.3 WT and H3.3 MUT cells analyzed in the manuscript were obtained from different patients or indicated as relapse samples by addition of ‘.2’ to the UPI.

### Isolation of GCTB stromal cells from patient tissue

Tumor tissue from surgical resections was mechanically cut into small pieces and digested with 1.5 mg/ml collagenase B (Roche Diagnostics, Mannheim, Germany) at 37 °C for 3 h in Dulbecco’s Modified Eagle Medium (DMEM) (Lonza GmbH, Wuppertal, Germany) high glucose supplemented with 10% fetal calf serum (FCS) (Biochrom, Berlin, Germany), and 100 U ml^−1^ penicillin/streptomycin (Lonza GmbH). Cells were collected by centrifugation, washed twice in PBS and cultured in DMEM as described above. Twenty-four hours after plating, cells were carefully treated with Trypsin/EDTA (Lonza GmbH) leaving the giant cells attached in the culture flask. Detached cells were cultured for further 2 passages to eliminate histiocytes and remaining giant cells.

### Isolation of nt-SC cell lines from the bone marrow

Nt-SCs were isolated from fresh bone marrow samples derived from the iliac crest under the approval of the Ethics Committee of the University of Heidelberg. Bone marrow cells were purified on a Ficoll-Paque™ Plus density gradient (GE Healthcare, München, Germany), washed in PBS and treated with erythrocyte lysis buffer (0.154 M NH_4_Cl, 10 mM KHCO_3_, 0.1 mM EDTA) to remove erythrocytes. The nt-SC-enriched fraction was seeded and cultured in DMEM high-glucose supplemented with 12.5% FCS (Biochrom), 2 mM L-glutamine, 100 U ml^−1^ penicillin, 100 μg ml^−1^ streptomycin (Lonza), 4 ng/ml basic fibroblast growth factor (Merck Chemicals GmbH, Darmstadt, Germany, 50 μM 2−mercaptoethanol and 1% non-essential amino acids (Invitrogen, Karlsruhe, Germany). After 48 h, cultures were washed with PBS to remove non-adherent material. During expansion, medium was replaced twice a week.

### Cell line maintenance

The GCTB stromal and nt-SC cell lines were cultivated in DMEM/F12 no phenol red (Gibco, Invitrogen, Life Technologies, Paislay, UK) supplemented with 10% FCS (Biochrom, Berlin, Germany) and 2 ng μl^−1^ basic FGF (Biolegend, San Diego, USA) at 37 °C and 5% CO_2_. To avoid contact inhibition, cells were split after reaching maximum 90% of confluency.

### Commercially available cell lines

All commercially available cell lines used within this work were authenticated: Hos143B: DSMZ 09/2018, full matching STR reference profile; CAL-72: COA Multiplexion 09/2018; identity based on a SNP-based assay 100%; HEK293T: COA Multiplexion 06/2020, identity based on a SNP-based assay 100%; HeLa: Korean Cell Line Bank, COA 03/2018, full matching STR reference profile.

### Isogenic cell lines

The establishment of H3.3-GFP isogenic cell lines in HeLa cells utilized the zinc-finger (ZF) targeting methodology^24^. Cells were transfected with the two zinc finger subunits-encoding vectors pAC HA nIL2RGL hNeeai_1140 and _1141 and the targeting constructs containing the cDNA sequence of *H3F3A* ligated into the pBluescript II (SK-) vector together with an in-frame eGFP locus and IRES separating Neo-selection cassette by NEPA21 (Nepa Gene Co. Ltd.) electroporation. Subsequently, the cells were cultured in media under neomycin selection using G418 for four weeks. Surviving cells were FACS-sorted and individual clones were allowed to propagate. HEK293T were lentivirally-transduced with human H3.3-HA-3XFLAG constructs in pLVX expression vectors and selected with puromycin. Cells were maintained in DMEM (Invitrogen™ Life Technologies, Carlsbad, USA) supplemented with 10% FCS (Biochrom, Berlin, Germany) and penicillin-streptomycin (Sigma). HEK293T were transduced with lentivirus produced using second generation lentiviral vectors (Plasmidfactory, Bielefeld, Germany), as well as pLVX transfer vectors in HEK293T cells. H3.3-HA-3XFLAG encoding pLVX vectors were obtained from BioCat GmbH for H3.3 wt, H3.3-G34A, H3.3-G34L, H3.3-G34R, and H3.3-G34W. Constructs encoding for H3.3-G34V, H3.3-K27M and H3.3-K36M were generated via site-directed mutagenesis following the manufacturers protocol (Q5^®^ Site-Directed Mutagenesis kit, New England BioLabs, USA).

### Immunohistochemistry

For immunohistochemical detection of the H3.3-G34W mutation formalin fixed paraffin embedded GCT tissue sections were deparaffinized in Roti-Histol (Carl Roth GmbH, Karlsruhe, Germany) and rehydrated in isopropanol. Antigen retrieval was performed using Dako target retrieval solution pH 6 (Dako, Hamburg, Germany) for 5 min at 121 °C in a pressure cooker. Sections were blocked for 30 min at room temperature using PBS supplemented with 5% bovine serum albumin (BSA). The primary rabbit anti-H3G34W antibody (Active Motiv, Carlsbad, USA) was diluted 1:1000 in PBS/1% BSA and incubated over night at 4 °C. The signal was amplified using the BrightVision +Poly-AP kit (VWR, Darmstadt, Germany) according to the manufacturer´s instructions. Samples were counterstained with hematoxylin (Carl Roth GmbH) and mounted using Neo-Mount (Merck, Darmstadt, Germany). All used antibodies are listed in Supplementary Table 1.

### Lineage verification by flow-cytometric analysis

Stromal cells were harvested by trypsination and transferred to 1,5 ml Eppendorf in PBS. Cells were washed with PBS 2% FCS and centrifuged for 5 min at 250×*g* at 4 °C. The antibody staining cocktail was prepared in PBS 2% FCS and cells were stained 20 min at 4 °C (all antibodies are specified in the Supplementary Table 1**;** dilutions: anti-CD45, anti-CD235, anti-CD105 1:50; anti-CD90 1:100). Afterwards cells were washed with PBS 2% FCS to remove unbound antibodies and centrifuged at 250×*g* for 5 min. To exclude dead cells during flow cytometric analysis, propidium iodide was added prior to flow analysis. Data were acquired on a BD FACSAria Fusion (Beckton, Dickinson and Company (BD)) and data were analyzed using FlowJo (BD). Prism (GraphPad Software) was used to generate bar graphs. Hematopoietic/erythroid cells were defined as CD45+CD235+, CD45−CD235− CD105+and CD45−CD235− CD90+ cells were defined as mesenchymal cells. Freshly frozen iliac crest bone marrow aspirates from healthy donors were used as healthy controls.

### OPG expression quantification with ELISA

5000 cells were seeded into a 96-well plate and cultivated in 100 µl media for two days. Enzyme-linked immunosorbent assay (ELISA) was performed with the Abcam human Osteoprotegerin ELISA Kit following the manufacturer´s instructions.

### Cell titer blue assay

Cells were cultivated in a 96-well plate. 100 µl fresh media (DMEM/F12 no phenol red (Gibco, Invitrogen, Life Technologies, Paislay, UK) supplemented with 10% FCS (Biochrom, Berlin, Germany) were added and supplemented with 20 µl CTB reagent from Promega. After 2.5 h of cultivation at 37 °C fluorescence was recorded at 560Ex/590Em.

### siRNA knockdown

120,000 cells were seeded into a 6-well plate. After 24 h, medium was changed and transfection was performed using 1 µl Dharmafect1 (Horizon Discovery, Waterbeach, UK) reagent in combination with 2 µl of a 25 µM siRNA SMARTpool of Dharmacon (Horizon Discovery, Waterbeach, UK) targeting *EBF2*. Cells were harvested 48 h after transfection.

### Detection of the H3F3A-G34W mutation by mutation-specific PCR

Genomic DNA was isolated using the Quick DNA Miniprep kit (Zymo research, Freiburg, Germany) according to the manufacturer’s protocol. PCR amplification was performed using *H3F3A* wild-type and *H3F3A*-G34W specific primer, respectively. The reaction consisted of 2 U Platinum Taq polymerase (Thermo Fisher Scientific, Dreieich, Germany), 0.6 µl MgCl_2_ (50 mM), 0.4 µl dNTPs (10 mM each), 0.5 µl of each primer (10 µM) and 100 ng genomic DNA as template in a total volume of 20 µl. Samples were incubated at 94 °C for 3 min followed by 34 cycles of denaturation at 94 °C for 15 s, annealing at 66 °C for 20 s and extension at 72 °C for 30 s and a final extension step at 72 °C for 7 min. PCR products were separated on a 1.6% agarose gel, visualized by Midori Green (Biozym, Hessisch Oldendorf, Germany) and imaged. All primers are listed in Supplementary Data 5.

### Detection of the H3F3A-G34W mutation by Sanger sequencing

DNA was extracted using the Qiamp Mini Kit (Qiagen, Hilden, Germany), according to the manufacturer´s instructions. The PCR reaction to amplify the mutation spanning *H3F3A* region consisted of 1U Hot Star Taq polymerase (Thermo Fisher Scientific, Dreieich, Germany), 0.8 µl dNTPs (10 mM each, Fermentas, St. Leon-Rot, Germany), 2 µl of each primer (10 µM, Sigma-Aldrich, Taufkirchen, Germany) and 100 ng genomic DNA as template in a total volume of 40 µl. Samples were incubated at 95 °C for 15 min followed by 10 cycles of denaturation at 94 °C for 45 s, annealing at 61–56 °C for 30 s (touchdown −0.5 °C/cycle) and extension at 72 °C for 60 s followed by 25 cycles of denaturation at 94 °C for 45 s, annealing at 56 °C for 30 s and extension at 72 °C for 60 s and a final extension step at 72 °C for 10 min. Sequencing was performed by GATC Biotech AG, Konstanz, Germany. All primers are listed in Supplementary Data 5.

### Deep targeted resequencing using MiSeq

Deep resequencing of *H3F3A* amplicons was performed according to^52^. DNA was extracted using the Qiamp Mini Kit (Qiagen, Hilden, Germany), according to the manufacturer´s instructions. The PCR reaction to amplify the mutation spanning region of the *H3F3A* gene consisted of 0.35U HotStart Q5 polymerase (NEB, Ipswich, USA), 0.6 µl dNTPs (10 mM each, Fermentas, St. Leon-Rot, Germany), 0.3 µl of each primer (10 µM, Sigma-Aldrich, Taufkirchen, Germany) and 25 ng genomic DNA as template in a total volume of 25 µl. Samples were incubated at 98 °C for 1 min followed by 33 cycles of denaturation at 98 °C for 10 s, annealing at 61 °C for 30 s and extension at 72 °C for 20 s, and a final extension step at 72 °C for 2 min. Primer sequences are listed in Supplementary Data 5. Primers included a sequence complementary to the primers used for library preparation. Samples were separated on a 1.2% agarose gel and DNA was visualized by Ethidium Bromide. Gel extraction was performed using the Gel extraction Kit (Qiagen, Hilden, Germany). Libraries were prepared using 12.5 µl NEB Next HF 2x PCR mix (New England Biolabs, USA) in combination with 0.75 µl of 10 µM IDT primers with Nextera handles and TruSeq Unique-Dual Indices (IDT, USA), 0.3 µl 100× Sybr green and 11 µl of DNA (2 ng). Amplification was performed for 6 cycles with the following progression: 98 °C 30 s, 98 °C 10 s, 62 °C 30 s, and 72 °C 15 s. Libraries were pooled and sequenced on a single flow-cell lane in a or 300 bp paired-end MiSeq run (Illumina, San Diego, CA, USA). The reads were demultiplexed using custom scripts, aligned to the GRCh37 assembly using *bwa mem* (v. 0.7.8)^53^ with default settings. Base frequencies were read into R and analyzed using package *deepSNV* (v. 1.24.0)^54^.

### Detection of genomic structural variations using M-FISH

Multiplex fluorescence in situ hybridization (M-FISH) was performed as described in^55^. Seven pools of flow-sorted whole chromosome painting probes were amplified and directly labeled by degenerative oligonucleotide primed (DOP)-PCR using CY-415-aadUTP (Dyomics, Jena, Germany), Green-dUTP (Abbott, catalog number 02N32-050), DY-547P1-aadUTP (Dyomics, Jena, Germany), DY-590-aadUTP (Dyomics, Jena, Germany), and CY-647P1-aadUTP (Dyomics, Jena, Germany) conjugated nucleotides or biotin-16-dUTP (Roche Diagnostics, Mannheim, Germany) and digoxigenin-11-dUTP (Roche Diagnostics, Mannheim, Germany), respectively. Prior hybridization, metaphase preparations of the H3.3 WT and H3.3 MUT cells were digested with pepsin (0.5 mg/ml; Sigma-Aldrich, USA) in 0.2 N HCL (Carl Roth GmbH, Karlsruhe, Germany) for 10 min at 37 °C, washed in PBS, post-fixed in 1% formaldehyde, dehydrated with a degraded ethanol series and air dried. Slides were denatured in 70% formamide/1× SSC/15% dextran sulfate for 2 min at 72 °C. Hybridization mixture consisting of 50% formamide, 2× SSC, Cot1-DNA, and labeled DNA probes was denatured for 7 min at 75 °C, preannealed for 20 min at 37 °C, and hybridized to the denatured metaphase preparations. After 48 h incubation at 37 °C slides were washed at room temperature in 2× SSC, 3 × 5 min, followed by 2 × 5 min in 0.2% SSC/0.2% Tween-20 at 56 °C. For indirect labeled probes, a immunofluorescence detection was carried out. Biotinylated probes were visualized using three layers of antibodies: (1) streptavidin Alexa Fluor 750 conjugate (dilution 1:100 in 4× SSC/0.2% Tween-20), (2) biotinylated goat anti avidin (dilution 1:200 in 4× SSC/0.2% Tween-20) (3) followed by a second streptavidin Alexa Fluor 750 conjugate (dilution 1:100 in 4× SSC/0.2% Tween-20). Digoxigenin labeled probe were visualized using rabbit anti digoxin (dilution 1:500 in 4 x SSC/0.2% Tween-20) followed by goat anti rabbit IgG Cy5.5 (dilution 1:500 in 4× SSC/0.2% Tween-20). All antibodies are summarized in Supplementary Table 1. Slides were washed in 4× SSC/0.2% Tween-20, counterstained with 4.6-diamidino-2-phenylindole (DAPI) and covered with antifade solution. Images of 20 metaphase spreads of the H3.3 WT and H3.3 G34W cells were captured for each fluorochrome using highly specific filter sets (Chroma technology, Brattleboro, VT), and processed using the Leica MCK software (Leica Microsystems Imaging Solutions, Cambridge, UK), respectively

### Chromatin fractionation

Cell pellets were resuspended in lysis buffer (10 mM HEPES pH 7.6, 10 mM KCl, 0.05% NP40) with protease inhibitors and incubated on ice for 30 min. Samples were centrifuged at 16,000×*g* at 4 °C for 10 min and the supernatant was taken as cytosolic fraction. Leftover pellet was further lysed with low salt buffer (10 mM Tris HCl pH 7.5, 3 mM MgCl_2_) supplemented with 1% Triton X-100 for 15 min on ice and centrifuged. Supernatant was taken as nuclear proteins. Leftover pellet was resuspended in 0.2 M HCl, incubated on ice for 20 min and centrifuged. The supernatant was neutralized with 1 M Tris HCl buffer (pH8) and used as the chromatin fraction.

### Western blot

Proteins were separated using SDS polyacrylamide gel electrophoresis (SDS-PAGE) and analyzed by Western blotting using the antibodies listed in the Supplementary Table 1. Antibody dilutions: anti-β-actin 1:5000, anti-H3.3 1:250, anti-H3.3-G34W 1:500, anti-H3K36me2 1:500, anti-HK36me3 1:500, anti-H4 1:500, anti-DAXX 1:1000, anti-α-tubulin 1:2000, anti-FLAG 1:1000. Chemiluminescence signals were imaged using Amersham Imager 680 (GE, Boston, USA). For the separation of endogenous histone H3 proteins and ectopically-expressed H3.3-HA-3XFLAG in HEK293T, we used 8–16% Mini-PROTEAN® TGX gels (Bio-Rad Laboratories).

### Differentiation and alkaline phosphatase staining

50.000 cells were seeded in 24-well plates. When confluent medium was changed to osteogenic differentiation medium: DMEM high Glucose mit L-Glutamin supplemented with 10%FCS, 0.1 µM Dexamethason, 0.17 mM Ascorbinsäure-2-phosphat, and 10 mM ß-Glycerophosphat. Alkaline phosphatase activity was analyzed using BCIP/NBT Alkaline Phosphatase Substrate Kit (Vector Laboratories, Burlingame, CA, USA) after 1 min of 4% PFA fixation.

### Quantification of ALP activity

50,000 cells were seeded in 24-well plates. When confluent media was changed to differentiation media (compare Differentiation and alkaline phosphatase staining) and the first measurement was performed. After CTB as described above, wells were PBS washed and fixated for 60 s with 4% PFA in PBS. After PBS washing 500 µl Alkaline Phosphatase Yellow (pNpp) Liquid Substrate (System for ELISA, Sigma-Aldrich, USA) was added and incubated at 37 °C for 8 min. Absorbance was measured at 405 nm.

### Whole-genome sequencing and analysis

Read pairs were mapped to the human reference genome (build 37, version hs37d5), using *bwa mem* (v. 0.7.8)^53^ with minimum base quality threshold set to zero [-T 0] and remaining settings left at default values, followed by coordinate-sorting with *bamsort* (with compression option set to fast REF1) and marking duplicate read pairs with *bammarkduplicates* (with compression option set to best^56^); both are part of *biobambam* package (v.0.0.148)^57^. Somatic SNVs were identified with the DKFZ SNV-calling workflow^58^. Somatic SNVs and indels in matched tumor normal pairs were identified using the DKFZ core variant calling workflows of the ICGC Pan-cancer Analysis of Whole Genomes (PCAWG) project (https://dockstore.org/containers/quay.io/pancancer/pcawg-dkfz-workflow). Tumor and matched control samples were analyzed by *Platypus* (v. 1.0)^59^ to identify indel events. SNVs and indels from all samples were annotated using *ANNOVAR* (v. 2017Jul16)^56^ according to GENCODE gene annotation (v. 19) and overlapped with variants from dbSNP10 (build 141) and the 1000 Genomes Project database. Genomic structural rearrangements were detected using *SOPHIA* (v.34.0) (https://bitbucket.org/utoprak/sophia/src) as described in ref. ^60^. Briefly, *SOPHIA* uses supplementary alignments as produced by *bwa mem* as indicators of a possible underlying SV. SV candidates are filtered by comparing them to a background control set of sequencing data obtained using normal blood samples from a background population database of 3261 patients from published TCGA and ICGC studies and both published and unpublished DKFZ studies, sequenced using Illumina HiSeq 2000 (100 bp), 2500 (100 bp), and HiSeq X (151 bp) platforms and aligned uniformly using the same workflow as in this study. An SV candidate is discarded if (i) it has more than 85% of read support from low quality reads; (ii) the second breakpoint of the SV was unmappable in the sample and the first breakpoint was detected in 10 or more background control samples; (iii) an SV with two identified breakpoints had one breakpoint present in at least 98 control samples (3% of the control samples); or (iv) both breakpoints have less than 5% read support. Statistics over SVs for 9 samples with matched control and integrated variant analysis over all samples were based on *SOPHIA* calls. Allele-specific copy-number aberrations were detected using *ACEseq* (v. 1.0)^61^. SVs called by *SOPHIA* were incorporated to improve genome segmentation.

### Whole-genome sequencing data of bone cancer and glioblastoma

WGS data of 73 bone cancer patients (BOCA cohort) were obtained via the Pan-cancer Analysis of Whole Genomes Project^62^. Pediatric glioblastoma data (PGBM cohort) were obtained from PedBrain Tumor Project of the International Cancer Genome Consortium^20^. The processing and variant calling (SNVs, SVs, CNVs) was performed consistently with the GCTB data as described above. To balance sample sizes for the analysis of recurrent genetic events, in addition to analyzing the complete BOCA cohort (Supplementary Fig. 1i), we draw several random 10-patient subsets, one of which is shown in Fig. 1d. From the PGBM cohort only 10 samples with H3.3-G34R mutation were selected for the analysis. Patient identifiers of all WGS samples are given in Supplementary Data 1.

### ATAC-sequencing and analysis

Libraries for ATAC-sequencing were prepared in accordance with the original protocol with minor modifications^63^. 50,000 cells were lysed by 1% NP40 and PBS washed. After centrifugation at 1200×*g* for 10 min, tagmentation at 55 °C for 8 min in a reaction mix with 2.5 µl of TDE1 (Nextera Illumina DNAKit), 25 µl Tagmentation buffer (Nextera Illumina DNA Kit) and 22 µl water took place. Reaction was stopped by adding 10 µl Guanidium (5 M) and samples were purified using 72 µl Ampure Beads. Libraries were generated using NEBNext High Fidelity PCR Mix and sequenced on the Illumina HiSeq 2000 platform. Sequencing reads were adapter-trimmed using *cutadapt* (v. 1.10)^64^. Genomic alignments were performed against the human reference genome (hg19, NCBI build 37.1) using *Bowtie2* (v. 2.3.0)^65^. The non-default parameters *-q 20 -s* were used. PCR duplicates were removed by *Picard MarkDuplicates* (v. 1.125). Signal tracks were generated using *deepTools* (v. 2.3.3)^66^. A compatible CWL-based ATAC-seq data processing workflow is available at https://github.com/CompEpigen/ATACseq_workflows. Peaks were called using *Macs2* (v. 2.1.1.)^67^ with the parameters *–nomodel –shift -50 –extsize 100 –qvalue 0.01*. All peaks were merged to create a common bed file with read counts before differential analysis using *edgeR* (v. 0.3.16)^68^. Gene annotations were made using *ChIPpeakAnno* (v. 3.18.0)^69^. Transcription binding motif analysis was performed using *HOMER* (v. 4.9)^70^. Motifs with a *P*-value <0.01 and a ratio of motif to background above 1.1 were defined as significantly enriched.

### ChIP-sequencing and analysis

We used the ChIP-mentation protocol^71^ to map the genomic distribution of WT and G34W H3.3, total H3, as well as 6 histone modifications (H3K4me1, H3K4me3, H3K9me3, H3K27ac, H3K27me3, and H3K36me3) in a subset of samples (see details in Supplementary Data 1). To confirm the specificity of the H3.3 and H3.3 G34W antibody used for ChIP-Seq analysis, we performed a validation experiment with a Histone code peptide array (JPT, Berlin, Germany) containing short peptides that densely cover most of the known histones and their modifications. We did not observe any significant binding of the G34W-speicifc antibody to H3.3 peptides and a highly specific binding of the H3.3 antibody. Cells of a confluent T175 flask were harvested by TrypLE incubation After centrifugation for 5 min at 1000 xg, cells were washed in PBS, distributed into 750.000 cell aliquots and pelleted again. The cell pellet fixed in 8 ml 1% FA in PBS at room temperature for 10 min. Crosslinking was stopped by the addition of 400 μl 2.5 M glycine. Cells were pelleted, resuspended in 1 ml ice cold PBS supplemented with 1x protease inhibitor and again pelleted at 3000×*g* for 3 min at 4 °C. The supernatant was taken off and the pellet was resuspended in 130 μl FL buffer supplemented with 2× protease inhibitor and transferred to an AFA Covaris 130 μl tube. For nuclei isolation, cells were sheared with a duty factor of 2.5, 200 burst and 40 watts for 3–10 min using the Covaris M220. The nuclei were spinned down for 3 min at 4 °C. The nuclei pellet was resuspended in 130 μl shearing buffer and shearing with a duty factor of 5, 75 watt and 200 burst was performed for 10–12 min with the Covaris M220. 100-200 ng sheared DNA were filled up to 200 μl with dilution buffer and mixed with 1 µg antibody and rotated over night at 4 °C. The storage solution of 20 μl Protein A beads per IP was taken off using a magnet and the beads were washed with 500 μl PBS supplemented with 0.1% BSA. To block the beads, they were resuspended in 150 μl PBS with 0.1% BSA and also rotated over night at 4 °C. The next day, the PBS was taken off from the beads with the help of a magnet and beads were taken up in 20 μl dilution buffer. The bead-dilution buffer mixture was added to the sheared DNA-antibody mixture and rotated for 2 h at 4 °C. The supernatant of the mixture was taken off with the help of a magnet and the bead-bound chromatin antibody conjugate was washed twice with 500 μl WB1, once with WB2 and once with WB3. Two more washing steps with 10 mM Tris HCl ph8 took place. Beads were then resuspended in 30 μl tagmentation mix (15 μl tagmentation buffer, 14 μl water, 1 μl TDE1 (Nextera Illumina DNA Kit)) and incubated for exactly 10 min at 37 °C. The tagmentation mix was removed and beads were washed twice with 500 μl Tn5 buffer. DNA elution took place by incubation of the beads in 100 μl ChIP-mentation elution buffer supplemented with 2 μl proteinase K for 2 h at 55 °C and 8 h at 65 °C. The supernatant containing the DNA was mixed with 200 μl AMpure beads for purification. For elution, beads were mixed with 26 μl water and incubated for 3 min at room temperature. Beads were collected with a magnet and the supernatant was collected as it contained the DNA with added adapters. Barcode amplification was performed with 24 μl sample. 0.8 μl universal Tn5 fwd primer and 0.8 μl barcode primer, both complementary to the transposase added adapters, as well as 25 μl NEB Next High Fidelity 2× mix and 0.3 μl 100× Sybr green. Samples were incubated at 72 °C for 5 min for gap repair followed by 30 s incubation at 98 °C. Cycles of denaturation at 98 °C for 10 s, annealing for 30 s at 63 °C and extension at 72 °C for 30 s were repeated until the amplification curve almost reached saturation but for maximum 16 cycles. 70 μl AMpure beads were added to the PCR mixture for purification. For elution, beads were mixed with 20 μl elution buffer and incubated for 3 min at room temperature. Beads were collected with a magnet and the supernatant was collected as it contained the library. The detailed composition of all buffers is described in the original publication. Up to 4 libraries were equimolarly pooled and sequenced in a 125 bp paired end run on a HiSeq machine. Sequence reads were preprocessed using *cutadapt* (v. 1.10)^64^ and aligned with *Bowtie2* (v. 2.3.0)^65^ with the default command line options. A compatible CWL-based ChIP-seq data processing workflow is available at https://github.com/CompEpigen/ChIPseq_workflows. We used *deepTools* (v. 2.3.3)^66^ with non-default options *–binSize 10 –extendReads 400 –normalizeTo1x 2451960000 –ignoreForNormalization chrX* to quantify genomic coverage in fixed-size window intervals for meta-plots and heatmaps.

### Calling of H3.3-G34W enriched regions

We used the Poisson test-based binarization module of the ChromHMM software (v. 1.18)^72^ to generate 200 bp windows with statistically significant enrichment of the wild-type H3.3 or H3.3-G34W signal over a simulated background. Adjacent windows were merged to generate primary enrichment regions. The regions were filtered against a union of the ENCODE ChIP-seq blacklists.

### Whole-genome bisulfite sequencing and analysis

WGBS Libraries were prepared using the TruSeq DNA PCR-Free LT Library Preparation Kits with partially modified steps in fragmentation and bead clean up/size selection. Briefly, 2 µg genomic DNA (diluted in nuclease-free water) was fragmented to 150–200 bp using a Covaris ultrasonicator (Covaris, Inc.) and quality checked using Agilent TapeStation (Agilent Technologies). The fragmented DNA sample was diluted with water and split into two aliquots of ~1 µg in 50 µl, end-repaired and purified using 1.6X sample purification beads. Adenylation of 3′ ends and ligation of TruSeq LT adapters were performed as described in manufactures protocol. Then, adapter-ligated fragment libraries were treated with bisulfite using the EpiTect Bisulfite Kit (Qiagen) following the instructions in the Illumina WGBS for Methylation Analysis Guide. After bisulfite conversion the fragment libraries were enriched with 8 cycles of PCR using KAPA HiFi Uracil+ DNA Polymerase with customized primer (Supplementary Data 5) and an annealing temperature of 69 °C according to the settings for PE libraries in the technical Data Sheet (KAPA HiFi HotStart Uracil+ Ready Mix, KR0413-version 1.12, peqlab). Amplified libraries were purified with 1× Agencourt AMPure XP beads (Beckman Coulter Inc.) and quantified using Qubit fluorometer (Life Technologies-Invitrogen). Then both aliquots of one sample were pooled, validated using Agilent TapeStation and again quantified using Qubit fluorometer. The final libraries were pooled and clustered on the cBot (Illumina) according to the manufacturer’s instructions with a final concentration of 250 pM, spiked with 5% PhiX control v3. Paired-end 150 bp sequencing on HiSeq X was performed using standard Illumina protocols. Basic statistics about the sequencing results is given in Supplementary Data 1. Raw reads were processed using *Trimmomatic* (v. 0.36)^73^ and aligned against reference sequence of the Genome Research Consortium (v. 37) using *bwameth* (https://github.com/brentp/bwa-meth) wrapping *bwa mem* (v. 0.7.8)^53^ with default parameters, except for invoking*-T 0*. After alignment duplicates were marked by applying *Picard MarkDuplicates* (v. 1.125). Methylation calling was performed with *MethylDackel* (v. 0.3.0). A compatible CWL-based data processing workflow for bisulfite sequencing is available at https://github.com/CompEpigen/WGBS_workflows (BWA_meth_start_with_trimmed.cwl). Subsequently and prior to DMR calling, *BSmooth* was used (v. 1.4.0) with default parameters to smooth the processed methylation profiles in all samples^74^. We then used we *DSS* (v. 2.27.0)^75^ to call DMRs for pairwise comparison between H3.3 WT and H3.3 MUT cells. Regions with at least 3 CpGs, a minimum length of 50 bp and a Benjamin-Hochberg corrected *P* value <0.05 were selected. All DMRs were filtered requiring a minimal mean methylation-value difference of 0.1.

### HumanMethylation450 and HumanMethylationEPIC analysis

Methylation analysis using HumanMethylation450 arrays was performed by the Genomics and Proteomics Core Facility according to the manufacturer’s instructions. Profiling of isogenic HeLa cell lines with HumanMethylationEPIC arrays was conducted at Korean Cancer Center according to the manufacturer’s instructions. Unnormalized signals (IDAT files) were loaded into R using RnBeads software (v. 2.2.0)^76^ and subjected to preprocessing with default option settings. 10,000 sites most variable across all samples were used for both, Principal Component Analysis and clustering analysis, visualized as a heatmap.

### RNA-sequencing and analysis

Poly-A RNA sequencing of GCTB samples was performed according to the standard protocol^77^. Total RNA was prepared for each cell line by using the RNeasy Mini kit (Qiagen, Hilden, Germany) and library preparation was done using TruSeq Stranded mRNA Kit (Illumina), according the manufacturer’s instruction. Paired-end 125 bp sequencing runs were performed on Illumina Hiseq2000 v4 machines. Raw sequence reads were preprocessed using *cutadapt* (v. 1.10)^64^ to remove sequencing primers and adapters. Reads were aligned to the GRCh37 human reference genome with *HISAT2* (v. 2.0.4)^78^ with additional non-default parameters *–max-intronlen 20000 –no-unal –dta*. Transcripts were assembled and quantified with *StringTie* (v. 1.3.3)^79^ with the GRCh37 transcript database. The RNA-seq processing workflow is available from https://github.com/CompEpigen/RNASeq_GCTB. Differential expression analysis was performed using *DeSeq2* (v. 1.18.1)^80^. Genes were called differentially expressed at FDR 0.05 and the absolute log-fold difference of greater or equal to one.

### RNA-Seq of the differentiation samples

RNA-seq of the MSC differentiation samples was performed according to the following protocol. 50,000 cells were seeded in 6-well plates in maintenance medium: MesenPRO-RS™ (Thermo Fisher Scientific, Massachusetts, USA). After 3 days, medium was replaced with osteogenic differentiation medium: DMEM high glucose supplemented with 10% FBS, 1× non-essential amino acids (NEAA), 2 mM L-glutamine, 0.28 mM ascorbic acid, 10 mM β glycerophosphate, and 10 nM dexamethasone (Sigma-Aldrich, USA). At each time-point: 0, 0.5, 1, 2, 4, 6, 8, 12, 16, 24, 48, 72, 125, 168, 336, and 504 h during osteogenesis, total RNA was isolated using TRIzol (Invitrogen™ Life Technologies, Carlsbad, USA) with the Direct-zol RNA kit (ZymoResearch, USA) according to the manufacturer’s instruction. RNA-seq library preparation was carried out using the NEBNext Poly(A) mRNA Magnetic Isolation Module and NEBNext Ultra Directional RNA Library Prep Kit for Illumina (New England Biolabs, USA) according to the manufacturer’s instruction. The quantity and quality of the cDNA library were assessed using the Agilent 2200 tapestation (Agilent Technologies, Santa Clara, USA). Paired-end sequencing of the pooled library was carried out using the Illumina NextSeq 500 v2 kit (Illumina, San Diego, USA) according to the manufacturer’s instruction.

### qRT-PCR expression analysis

Total RNA was extracted using the RNeasy Mini kit (Qiagen, Hilden, Germany). cDNA was synthesized using random hexamers (Qiagen, Hilden, Germany), and Superscript III Reverse Transcriptase (Invitrogen, Life Technologies, Paislay, UK) according to the manufacturer’s instructions. qPCR mixture consisted of 3.5 µl Light Cycler 480 Probe master (Roche Diagnostics, Mannheim, Germany) 1 µl 10 µM Primer mix (Sigma-Aldrich, Taufkirchen, Germany) and 0.05 µl UPL probe (Roche Diagnostics, Mannheim, Germany). 2.5 µl of a 1:10 dilution of cDNA served as template. Expression analysis was performed on the LightCycler 480-2 (Roche) system with the following progression: 10 min 95 °C and 45 cycles of 10 s 95 °C, 20 s 55 °C, 1 s 72 °C. Alternative to the UPL system, Sybr green qPCR was performed in a total volume of 10 µl including 5 µl Prima Quant Mix (Steinbrenner, Wiesenbach, Germany), 0.6 µl Primermix (10 µM each, Sigma-Aldrich) and 2 µl of 1:10 dilution of the template cDNA. Samples were incubated at 95 °C for 15 min and 15 s at 95 °C, 30 s at 55 °C, and 10 s at 72 °C for 45 cycles. Target gene expression was normalized to the housekeeping gene GAPDH using the ΔCT method (relative expression is equal to 2^-ΔCT^). All used primers are listed in Supplementary Data 5.

### Targeted DNA methylation analysis using MassARRAY

Bisulfite treatment was performed with the EZ DNA Methylation Kit from Zymo Research following the manufacturer’s protocol. PCR was performed with primers listed in Supplementary Data 5 using the Qiagen HotStar Taq (Qiagen, Hilden, Germany). The Shrimp alkaline phosphatase step and in vitro transcription were performed with the EpiTYPER Reagent Set from Agena Bioscience following the manufacturer’s protocol. Analyses were performed on the Sequenom Platform (Agena Bioscience, San Diego, USA).

### Gene and genomic feature annotations

Unless specified otherwise, Ensembl transcript and gene annotations were used for the GRCh37 assembly (build 87). List of bivalent genes in H3.3 WT was compiled by overlapping the consensus H3.3 WT peaks of H3K27me3 and H3K4me3 within 2 kb from a RefSeq TSS. A consensus list of bivalent genes in human ESCs was obtained from ref. ^81^. A list of PRC2 target genes was found in ref. ^82^. A comprehensive list of imprinted genes was obtained from ref. ^83^. Replication timing domains for the annotation of LMDs originates from Repli-Seq data of ref. ^84^, processed and publicly deposited by *RepliScan* package^85^. MSC-specific chromatin states were taken from the 15-state *ChromHMM* model^72^ for bone-marrow derived MSCs generated by the Roadmap Epigenomics consortium^21^ (sample E026).

### Identification of large-scale methylation domains

DNA methylation data was summarized in 20 kb tiling windows to eliminate the small-scale variability (e.g., related to CpG islands). The changepoints were then called using R package *changepoint* (v. 2.2.2). DNA methylation data was summarized in the obtained segments and the latter were clustered using standard hierarchical clustering resulting in 6 stable clusters. After clustering adjacent segments that belonged to the same cluster were merged. Two smallest clusters were removed since one contained less then 10 segments and the other one exclusively Y-chromosome segments. R code for LMD identification is available from https://github.com/lutsik/CP-LMDs. For gene density estimation UCSC hg19 human gene annotation (R package xDb.Hsapiens.UCSC.hg19.knownGene) were used and for each LMD segment the number of overlapping genes was divided by its length in megabases (Mb).

### Genomic overlap enrichment analysis

We tested the significance of overlap of differential ATAC-seq peaks, H3.3 G34W incorporation regions and other regions of interest with publicly available genomic annotations using *LOLA* (v. 1.8.0)^86^. In the case of ATAC peaks a union of all called peaks in H3.3 WT and H3.3 MUT was used as background to test enrichments at H3.3 G34W gained and lost peaks relative to each other. For H3.3 WT and G34W enrichment regions the background set consisted of regions called for wild-type H3.3 in H3.3 WT and H3.3 MUT groups, as well as the H3.3-G34W regions. We used the *LOLA* core database for most of the analyses. Enrichment of repeat elements was based on a custom *LOLA* database created using the UCSC Repeat Masker track (http://www.repeatmasker.org).

### Gene set overrepresentation and enrichment analysis

We used gene sets from the Molecular Signatures Database v. 6.2 (MSigDB)^87^ to test for the overrepresentation of DEGs or genes associated with differential ATAC peaks, and the gene set enrichment analysis (GSEA) of DEGs. Overrepresentation analysis was performed with the help of R package *GeneOverlap* (v. 1.14.0). GSEA was performed with *fgsea* package (v. 1.4.1)^88^ using 10,000 permutations.

### Reporting summary

Further information on research design is available in the Nature Research Reporting Summary linked to this article.

## Supplementary information

Supplementary Information

Description of Additional Supplementary Files

Supplementary Data 1

Supplementary Data 2

Supplementary Data 3

Supplementary Data 4

Supplementary Data 5

Reporting Summary

## Data Availability

All raw patient-derived sequencing data from WGS, WGBS, ATAC-seq, ChIP-seq, RNA-seq and deep targeted resequencing have been deposited in the European Genome-Phenome Archive (EGA) under restricted access with the accession code: EGAS00001003730. Processed sequencing data and microarray data have been deposited in ArrayExpress with the accession codes: E-MTAB-7184 (ChIP-Seq), E-MTAB-9512 (ATAC-Seq), E-MTAB-9513 (WGBS), E-MTAB-9515 (RNA-Seq). RNA-Seq data of the MSC osteogenic differentiation experiment are deposited at the Gene Expression Omnibus (GEO) with the accession code: GSE129036. We furthermore used gene sets from the Molecular Signatures Database v. 6.2 (MSigDB): https://www.gsea-msigdb.org/gsea/msigdb/download_file.jsp?filePath=/msigdb/release/6.2/msigdb_v6.2_files_to_download_locally.zip, ChromHMM states of human bone-marrow by ENCODE project (sample E026): https://egg2.wustl.edu/roadmap/data/byFileType/chromhmmSegmentations/ChmmModels/coreMarks/jointModel/final/E026_15_coreMarks_segments.bed, Replication timing segments by Repliscan project (Hansen et al., 2010): https://de.cyverse.org/anon-files/iplant/home/gzynda/public/hansen2010_replicate/repliscan_50kb.gff3, Consensus list of ESC-specific bivalent genes^81^: http://www.oncotarget.com/index.php?journal=oncotarget&page=article&op=downloadSuppFile&path%5B%5D=13746&path%5B%5D=21048 LOLA Core database of functionally annotated genomic regions: http://big.databio.org/regiondb/LOLACore_180423.tgz. Database of common variant calls from the 1000 Genomes Project: http://ftp.1000genomes.ebi.ac.uk/vol1/ftp/release/20130502/. GRCh37 transcript database ftp://ftp.ncbi.nlm.nih.gov/refseq/H_sapiens/annotation/GRCh37_latest/refseq_identifiers/GRCh37_latest_genomic.gff.gz Repeat masker database: http://www.repeatmasker.org Source data are provided with this paper.

## Source data

Source Data

## Code availability

All presented results were obtained with the use of published and publicly available software tools introduced in the Methods section. Essential custom code is available from dedicated code repositories specified in the descriptions of respective computational methods above. DKFZ/PCAWG WGS analysis workflow is available as a Docker container from: https://dockstore.org/containers/quay.io/pancancer/pcawg-dkfz-workflow. Compatible CWL workflows for WGBS, ATAC-Seq and ChIP-Seq data processling are publicly available on Github/CompEpigen: https://github.com/CompEpigen/WGBS_workflows (BWA_meth_start_with_trimmed.cwl) https://github.com/CompEpigen/ATACseq_workflowshttps://github.com/CompEpigen/ChIPseq_workflows. A snakemake-based processing workflow for RNA-seq data is available at: https://github.com/CompEpigen/RNASeq_GCTB. Custom code for LMD calling using changepoint algorithms is available at: https://github.com/lutsik/CP-LMDs. Other custom code is available at https://github/lutsik/GCTB_Epigenome.

## References

[1] Mohammad F, Helin K (2017). Oncohistones: drivers of pediatric cancers. Genes Dev..

[2] Nacev BA (2019). The expanding landscape of ‘oncohistone’ mutations in human cancers. Nature.

[3] Bennett RL (2019). A mutation in histone H2B represents a new class of oncogenic driver. Cancer Discov..

[4] Szenker E, Ray-Gallet D, Almouzni G (2011). The double face of the histone variant H3.3. Cell Res..

[5] Goldberg AD (2010). Distinct factors control histone variant H3.3 localization at specific genomic regions. Cell.

[6] Voon HP, Wong LH (2016). New players in heterochromatin silencing: histone variant H3.3 and the ATRX/DAXX chaperone. Nucleic Acids Res..

[7] Banaszynski LA (2013). Hira-dependent histone H3.3 deposition facilitates PRC2 recruitment at developmental loci in ES cells. Cell.

[8] Bernstein BE (2006). A bivalent chromatin structure marks key developmental genes in embryonic stem cells. Cell.

[9] Schwartzentruber J (2012). Driver mutations in histone H3.3 and chromatin remodelling genes in paediatric glioblastoma. Nature.

[10] Behjati S (2013). Distinct H3F3A and H3F3B driver mutations define chondroblastoma and giant cell tumor of bone. Nat. Genet..

[11] Fang D (2016). The histone H3.3K36M mutation reprograms the epigenome of chondroblastomas. Science.

[12] Chan KM (2013). The histone H3.3K27M mutation in pediatric glioma reprograms H3K27 methylation and gene expression. Genes Dev..

[13] Lewis PW (2013). Inhibition of PRC2 activity by a gain-of-function H3 mutation found in pediatric glioblastoma. Science.

[14] Bender S (2013). Reduced H3K27me3 and DNA hypomethylation are major drivers of gene expression in K27M mutant pediatric high-grade gliomas. Cancer Cell.

[15] Lu C (2016). Histone H3K36 mutations promote sarcomagenesis through altered histone methylation landscape. Science.

[16] Shi L, Shi J, Shi X, Li W, Wen H (2018). Histone H3.3 G34 mutations alter histone H3K36 and H3K27 methylation in cis. J. Mol. Biol..

[17] Amanatullah DF, Clark TR, Lopez MJ, Borys D, Tamurian RM (2014). Giant cell tumor of bone. Orthopedics.

[18] Raskin KA, Schwab JH, Mankin HJ, Springfield DS, Hornicek FJ (2013). Giant cell tumor of bone. J. Am. Acad. Orthop. Surg..

[19] Goldring SR, Roelke MS, Petrison KK, Bhan AK (1987). Human giant cell tumors of bone identification and characterization of cell types. J. Clin. Invest..

[20] International Cancer Genome Consortium PedBrain Tumor P. (2016). Recurrent MET fusion genes represent a drug target in pediatric glioblastoma. Nat. Med..

[21] Roadmap Epigenomics C. (2015). Integrative analysis of 111 reference human epigenomes. Nature.

[22] Zhou W (2018). DNA methylation loss in late-replicating domains is linked to mitotic cell division. Nat. Genet..

[23] Moskovszky L (2009). Genomic instability in giant cell tumor of bone. A study of 52 cases using DNA ploidy, relocalization FISH, and array-CGH analysis. Genes Chromosomes Cancer.

[24] Lim J (2017). The histone variant H3.3 G34W substitution in giant cell tumor of the bone link chromatin and RNA processing. Sci. Rep..

[25] Balke M (2013). Denosumab treatment of giant cell tumour of bone. Lancet Oncol..

[26] Boyle WJ, Simonet WS, Lacey DL (2003). Osteoclast differentiation and activation. Nature.

[27] Kieslinger M (2005). EBF2 regulates osteoblast-dependent differentiation of osteoclasts. Dev. Cell.

[28] Boyce BF, Xing L, Chen D (2005). Osteoprotegerin, the bone protector, is a surprising target for beta-catenin signaling. Cell Metab..

[29] Wolock SL (2019). Mapping distinct bone marrow niche populations and their differentiation paths. Cell Rep..

[30] Zardo G (2002). Integrated genomic and epigenomic analyses pinpoint biallelic gene inactivation in tumors. Nat. Genet..

[31] Shapira SN (2017). EBF2 transcriptionally regulates brown adipogenesis via the histone reader DPF3 and the BAF chromatin remodeling complex. Genes Dev..

[32] Voon HP (2015). ATRX Plays a Key Role in Maintaining Silencing at Interstitial Heterochromatic Loci and Imprinted Genes. Cell Rep..

[33] Harutyunyan AS (2019). H3K27M induces defective chromatin spread of PRC2-mediated repressive H3K27me2/me3 and is essential for glioma tumorigenesis. Nat. Commun..

[34] Hardouin SN, Guo R, Romeo PH, Nagy A, Aubin JE (2011). Impaired mesenchymal stem cell differentiation and osteoclastogenesis in mice deficient for Igf2-P2 transcripts. Development.

[35] Xu JC, Wu GH, Zhou LL, Yang XJ, Liu JT (2016). Leptin improves osteoblast differentiation of human bone marrow stroma stem cells. Eur. Rev. Med. Pharm. Sci..

[36] Huang L, Teng XY, Cheng YY, Lee KM, Kumta SM (2004). Expression of preosteoblast markers and Cbfa-1 and Osterix gene transcripts in stromal tumour cells of giant cell tumour of bone. Bone.

[37] Lau CP (2017). Genome-wide transcriptome profiling of the neoplastic giant cell tumor of bone stromal cells by RNA sequencing. J. Cell Biochem.

[38] Zhang Y (2017). Molecular basis for the role of oncogenic histone mutations in modulating H3K36 methylation. Sci. Rep..

[39] Sturm D (2012). Hotspot mutations in H3F3A and IDH1 define distinct epigenetic and biological subgroups of glioblastoma. Cancer Cell.

[40] de la Rica L (2013). PU.1 target genes undergo Tet2-coupled demethylation and DNMT3b-mediated methylation in monocyte-to-osteoclast differentiation. Genome Biol..

[41] Rauch A (2019). Osteogenesis depends on commissioning of a network of stem cell transcription factors that act as repressors of adipogenesis. Nat. Genet..

[42] Lau YS, Sabokbar A, Gibbons CL, Giele H, Athanasou N (2005). Phenotypic and molecular studies of giant-cell tumors of bone and soft tissue. Hum. Pathol..

[43] Murata A, Fujita T, Kawahara N, Tsuchiya H, Tomita K (2005). Osteoblast lineage properties in giant cell tumors of bone. J. Orthop. Sci..

[44] Chiellini C (2008). Stathmin-like 2, a developmentally-associated neuronal marker, is expressed and modulated during osteogenesis of human mesenchymal stem cells. Biochem. Biophys. Res Commun..

[45] Fang HT (2018). Global H3.3 dynamic deposition defines its bimodal role in cell fate transition. Nat. Commun..

[46] Yuen BT, Bush KM, Barrilleaux BL, Cotterman R, Knoepfler PS (2014). Histone H3.3 regulates dynamic chromatin states during spermatogenesis. Development.

[47] Liu L (2014). Enrichment of c-Met+ tumorigenic stromal cells of giant cell tumor of bone and targeting by cabozantinib. Cell Death Dis..

[48] Wulling M, Delling G, Kaiser E (2003). The origin of the neoplastic stromal cell in giant cell tumor of bone. Hum. Pathol..

[49] Wierzbinska JA (2020). Methylome-based cell-of-origin modeling (Methyl-COOM) identifies aberrant expression of immune regulatory molecules in CLL. Genome Med..

[50] Baubec T (2015). Genomic profiling of DNA methyltransferases reveals a role for DNMT3B in genic methylation. Nature.

[51] Dhayalan A (2010). The Dnmt3a PWWP domain reads histone 3 lysine 36 trimethylation and guides DNA methylation. J. Biol. Chem..

[52] Souren NY (2016). Mitochondrial DNA variation and heteroplasmy in monozygotic twins clinically discordant for multiple sclerosis. Hum. Mutat..

[53] Li H, Durbin R (2009). Fast and accurate short read alignment with Burrows-Wheeler transform. Bioinformatics.

[54] Gerstung M (2012). Reliable detection of subclonal single-nucleotide variants in tumour cell populations. Nat. Commun..

[55] Geigl JB, Uhrig S, Speicher MR (2006). Multiplex-fluorescence in situ hybridization for chromosome karyotyping. Nat. Protoc..

[56] Wang K, Li M, Hakonarson H (2010). ANNOVAR: functional annotation of genetic variants from high-throughput sequencing data. Nucleic Acids Res..

[57] Tischler G, Leonard S (2014). biobambam: tools for read pair collation based algorithms on BAM files. Source Code Biol. Med..

[58] Jones DT (2013). Recurrent somatic alterations of FGFR1 and NTRK2 in pilocytic astrocytoma. Nat. Genet..

[59] Rimmer A (2014). Integrating mapping-, assembly- and haplotype-based approaches for calling variants in clinical sequencing applications. Nat. Genet..

[60] Sahm F (2017). Meningiomas induced by low-dose radiation carry structural variants of NF2 and a distinct mutational signature. Acta Neuropathol..

[61] Kleinheinz, K. et al. ACEseq-allele specific copy number estimation from whole genome sequencing. Preprint at https://www.biorxiv.org/content/10.1101/210807v1 (2017).

[62] Consortium ITP-CAoWG. (2020). Pan-cancer analysis of whole genomes. Nature.

[63] Buenrostro JD, Giresi PG, Zaba LC, Chang HY, Greenleaf WJ (2013). Transposition of native chromatin for fast and sensitive epigenomic profiling of open chromatin, DNA-binding proteins and nucleosome position. Nat. Methods.

[64] Martin M (2011). Cutadapt removes adapter sequences from high-throughput sequencing reads. EMBnet J..

[65] Langmead B, Salzberg SL (2012). Fast gapped-read alignment with Bowtie 2. Nat. Methods.

[66] Ramirez F, Dundar F, Diehl S, Gruning BA, Manke T (2014). deepTools: a flexible platform for exploring deep-sequencing data. Nucleic Acids Res..

[67] Zhang Y (2008). Model-based analysis of ChIP-Seq (MACS). Genome Biol..

[68] McCarthy DJ, Chen Y, Smyth GK (2012). Differential expression analysis of multifactor RNA-Seq experiments with respect to biological variation. Nucleic Acids Res..

[69] Zhu LJ (2013). Integrative analysis of ChIP-chip and ChIP-seq dataset. Methods Mol. Biol..

[70] Heinz S (2010). Simple combinations of lineage-determining transcription factors prime cis-regulatory elements required for macrophage and B cell identities. Mol. Cell.

[71] Schmidl C, Rendeiro AF, Sheffield NC, Bock C (2015). ChIPmentation: fast, robust, low-input ChIP-seq for histones and transcription factors. Nat. Methods.

[72] Ernst J, Kellis M (2017). Chromatin-state discovery and genome annotation with ChromHMM. Nat. Protoc..

[73] Bolger AM, Lohse M, Usadel B (2014). Trimmomatic: a flexible trimmer for Illumina sequence data. Bioinformatics.

[74] Hansen KD, Langmead B, Irizarry RA (2012). BSmooth: from whole genome bisulfite sequencing reads to differentially methylated regions. Genome Biol..

[75] Park Y, Wu H (2016). Differential methylation analysis for BS-seq data under general experimental design. Bioinformatics.

[76] Assenov Y (2014). Comprehensive analysis of DNA methylation data with RnBeads. Nat. Methods.

[77] Weischenfeldt J (2013). Integrative genomic analyses reveal an androgen-driven somatic alteration landscape in early-onset prostate cancer. Cancer Cell.

[78] Kim D, Langmead B, Salzberg SL (2015). HISAT: a fast spliced aligner with low memory requirements. Nat. Methods.

[79] Pertea M (2015). StringTie enables improved reconstruction of a transcriptome from RNA-seq reads. Nat. Biotechnol..

[80] Love MI, Huber W, Anders S (2014). Moderated estimation of fold change and dispersion for RNA-seq data with DESeq2. Genome Biol..

[81] Court F, Arnaud P (2017). An annotated list of bivalent chromatin regions in human ES cells: a new tool for cancer epigenetic research. Oncotarget.

[82] Bracken AP, Dietrich N, Pasini D, Hansen KH, Helin K (2006). Genome-wide mapping of Polycomb target genes unravels their roles in cell fate transitions. Genes Dev..

[83] Skaar DA (2012). The human imprintome: regulatory mechanisms, methods of ascertainment, and roles in disease susceptibility. ILAR J..

[84] Hansen RS (2010). Sequencing newly replicated DNA reveals widespread plasticity in human replication timing. Proc. Natl Acad. Sci. USA.

[85] Zynda GJ (2017). Repliscan: a tool for classifying replication timing regions. BMC Bioinform..

[86] Sheffield NC, Bock C (2016). LOLA: enrichment analysis for genomic region sets and regulatory elements in R and Bioconductor. Bioinformatics.

[87] Subramanian A (2005). Gene set enrichment analysis: a knowledge-based approach for interpreting genome-wide expression profiles. Proc. Natl Acad. Sci. USA.

[88] Sergushichev, A. An algorithm for fast preranked gene set enrichment analysis using cumulative statistic calculation. Preprint at https://www.biorxiv.org/content/10.1101/060012v2 (2016).

