# Supplementary material for: Globally altered epigenetic landscape and delayed osteogenic differentiation in H3.3-G34W-mutant giant cell tumor of bone
Source: Nat Commun. 2020 Oct 27;11:5414. doi: 10.1038/s41467-020-18955-y (PMC7591516; doi:10.1038/s41467-020-18955-y)
Supplement: Supplementary file 1 — Supplementary Information [file 41467_2020_18955_MOESM1_ESM.pdf]

Globally altered epigenetic landscape and delayed osteogenic differentiation in H3.3-G34W-mutant giant cell tumor of bone

Lutsik et al.

Supplementary Material

Supplementary Figures

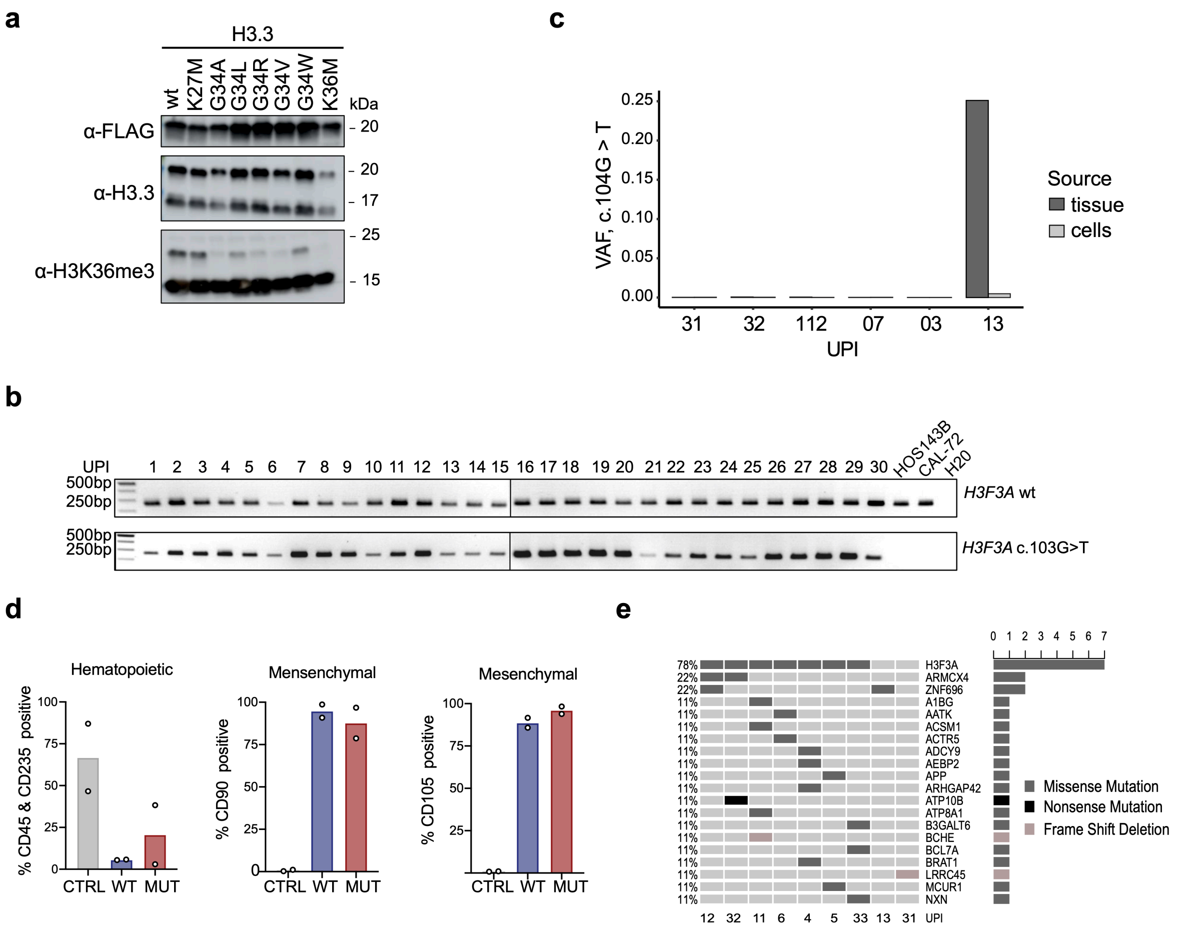

Supplementary figure 1: Initial characterization of GCTB samples

a. Western blots of whole cell lysates obtained from HEK293T cells stably expressing H3.3-HA-3XFLAG wildtype or respective mutants as indicated. The blot shown is representative of three independent replication experiments. **b**. Top: PCR for the wild-type *H3F3A* allele as control; bottom: mutation-specific PCR of the heterozygous *H3F3A* c.103G>T mutation in bulk DNA isolated from GCTB tumor resections. Osteosarcoma cell lines HOS143B and CAL-72 were used as negative controls. PCR assay cannot discriminate between c103G>T (G34W) and c103GG>TT (G34L) mutations. Vertical bar separates two independent gels. Size indicated in basepairs (bp). **c**. Quantification of the mutation at position 104 in the *H3F3A* gene (c.143G>T) leading to the H3.3 G34L substitution in tumor resections (dark grey) and derived stromal cell lines (light grey) using deep targeted resequencing. VAF, variant allele frequency. **d**. FACS analysis with CD45, CD235, CD90 and CD105 specific antibodies of two H3.3 WT (blue) and two H3.3 MUT stromal cell lines (red) (UPI: 13, 34, 20 und 21) in comparison to hematopoietic control cells (CTRL) from human bone marrow (grey). **e**. Most abundant single nucleotide variants (SNV) with driver potential based on whole-genome sequencing of nine patient-derived cell lines. **f.-i.** High-resolution version of circos plots shown in **Figure 1d-f.** Circos plot of the complete BOCA cohort (73 patients). Radial layers of bar plots represent the recurrence of selected classes of genomic variants across the cohorts. Green lines represent translocations, blue lines deletions, red lines duplications, and black lines inversions. The outermost layer represents functional small variants, namely SNVs and small indels that change the amino acid sequence of protein coding genes. The middle layer represents copy number variation across TADs. The inner layer represents SV recurrence across TADs. For random

subsets of the BOCA (n=10) cohort and the PGBM (n=10) and GCTB (n=9) cohorts, all layers have been scaled to the cohort size for maximum recurrence. For the entire BOCA cohort, the recurrence is scaled to the maximum recurrence encountered in all recurrence layers (40).

f

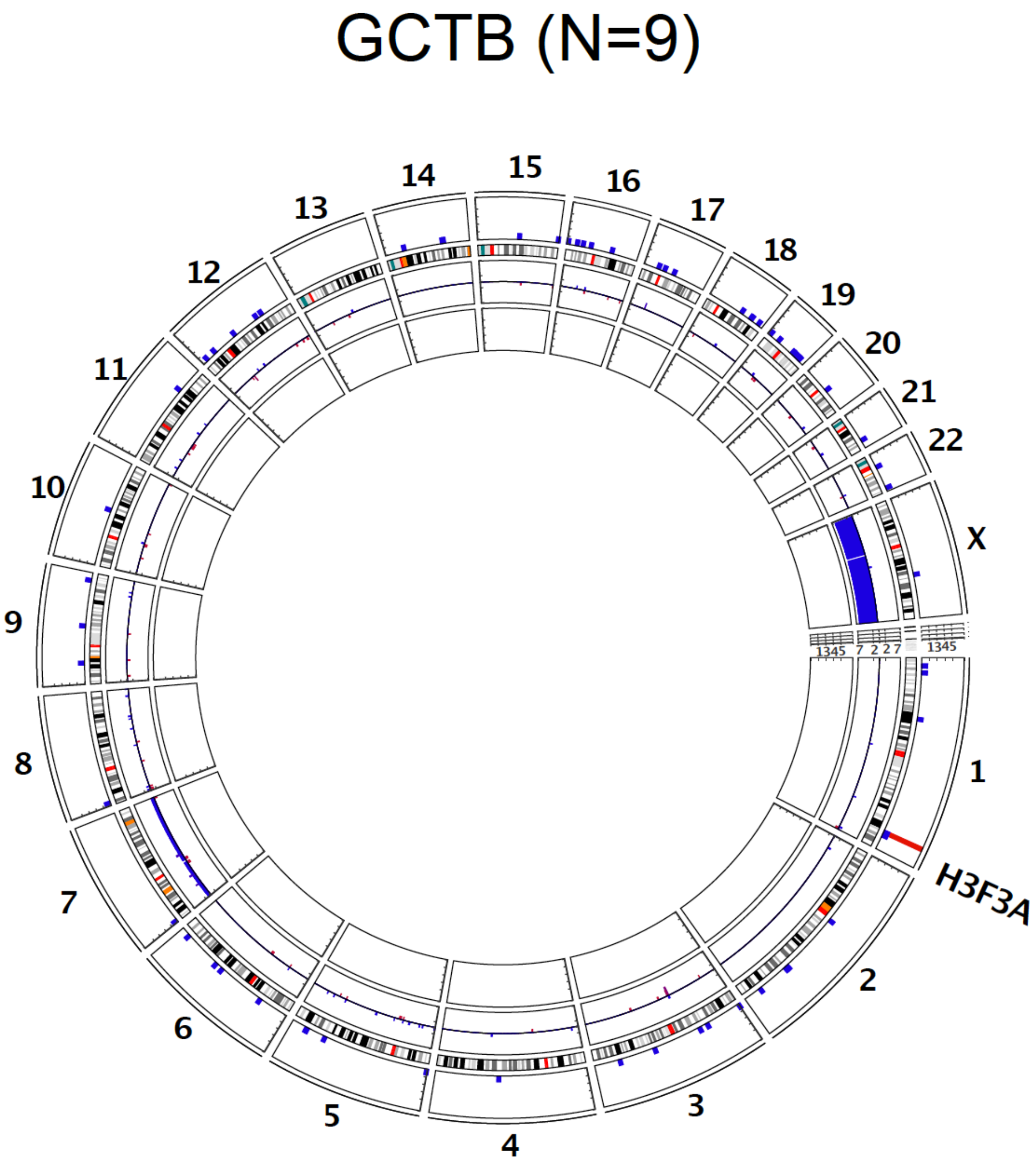

Supplementary figure 1 (continued)

g

G34-mutant PGBM (N=10)

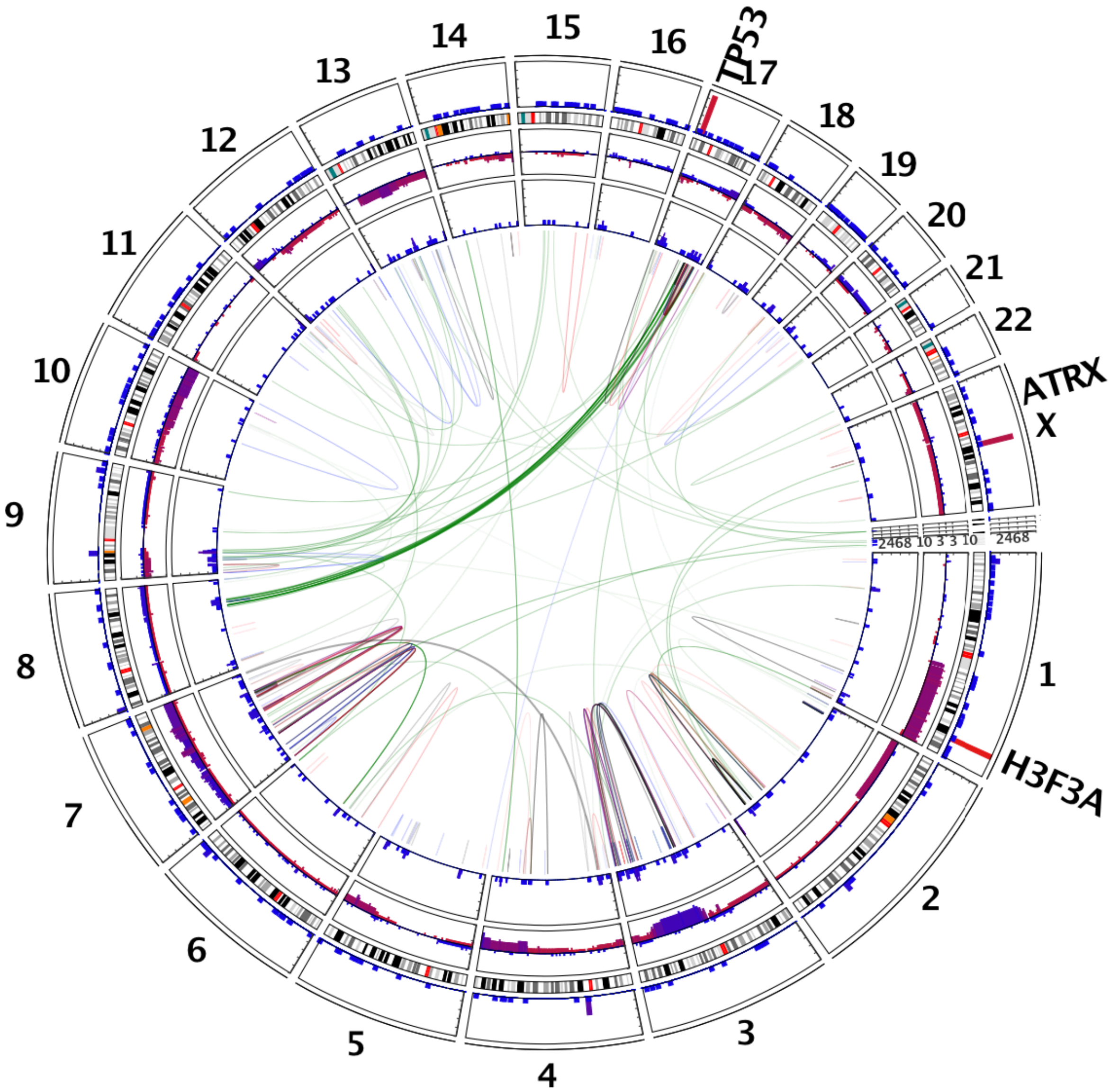

Supplementary figure 1 (continued)

h

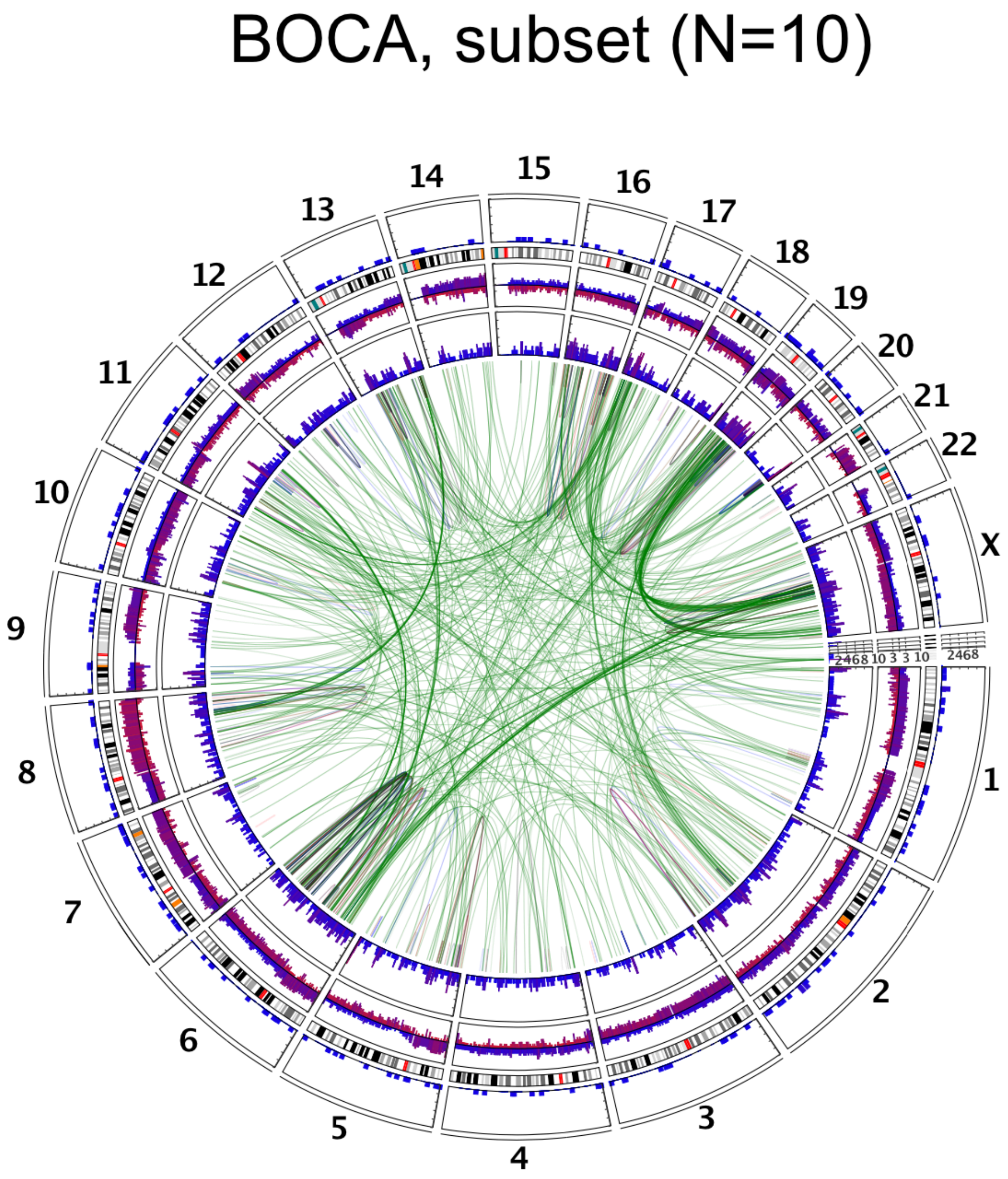

Supplementary figure 1 (continued)

i.

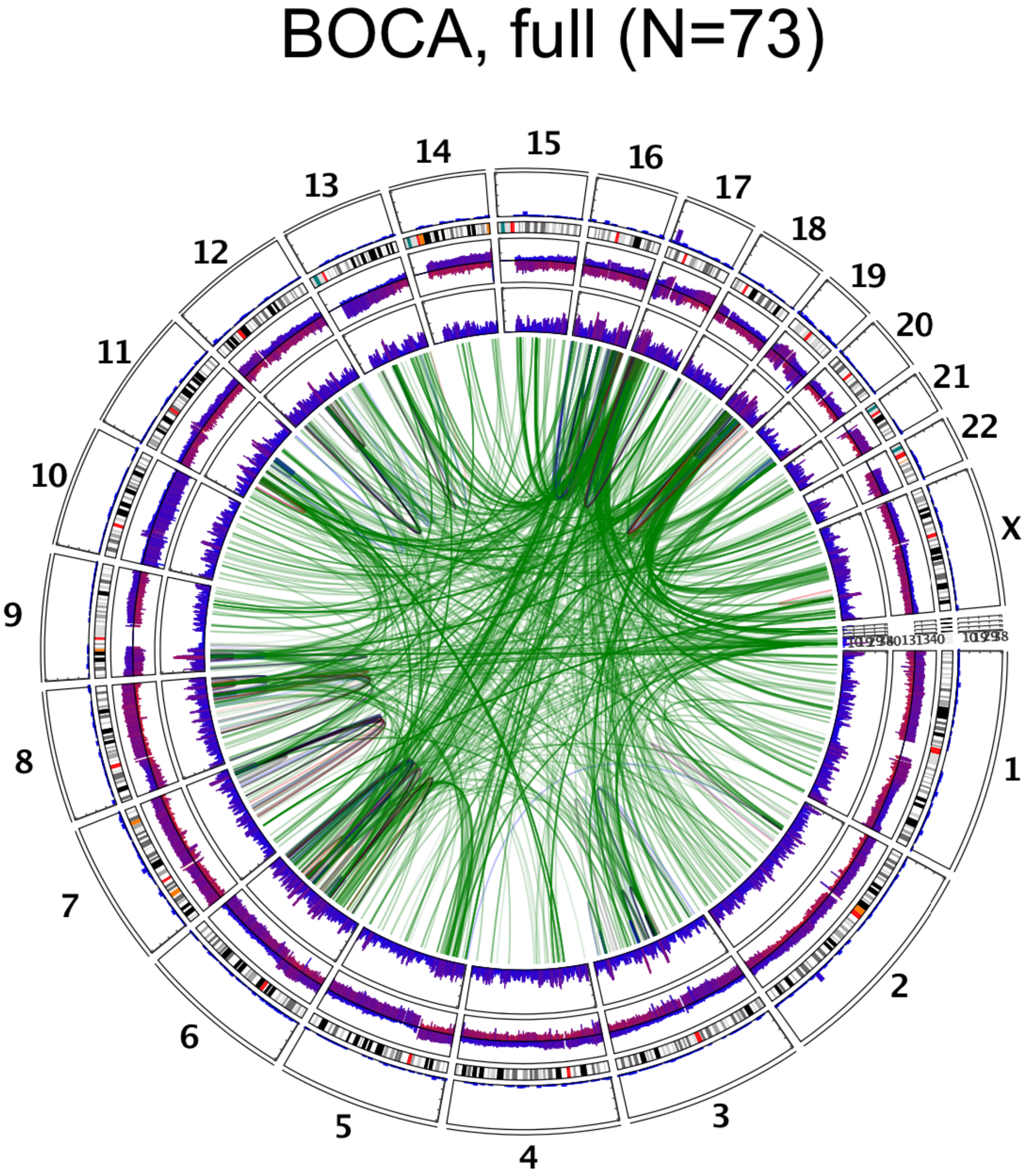

Supplementary figure 1 (continued)

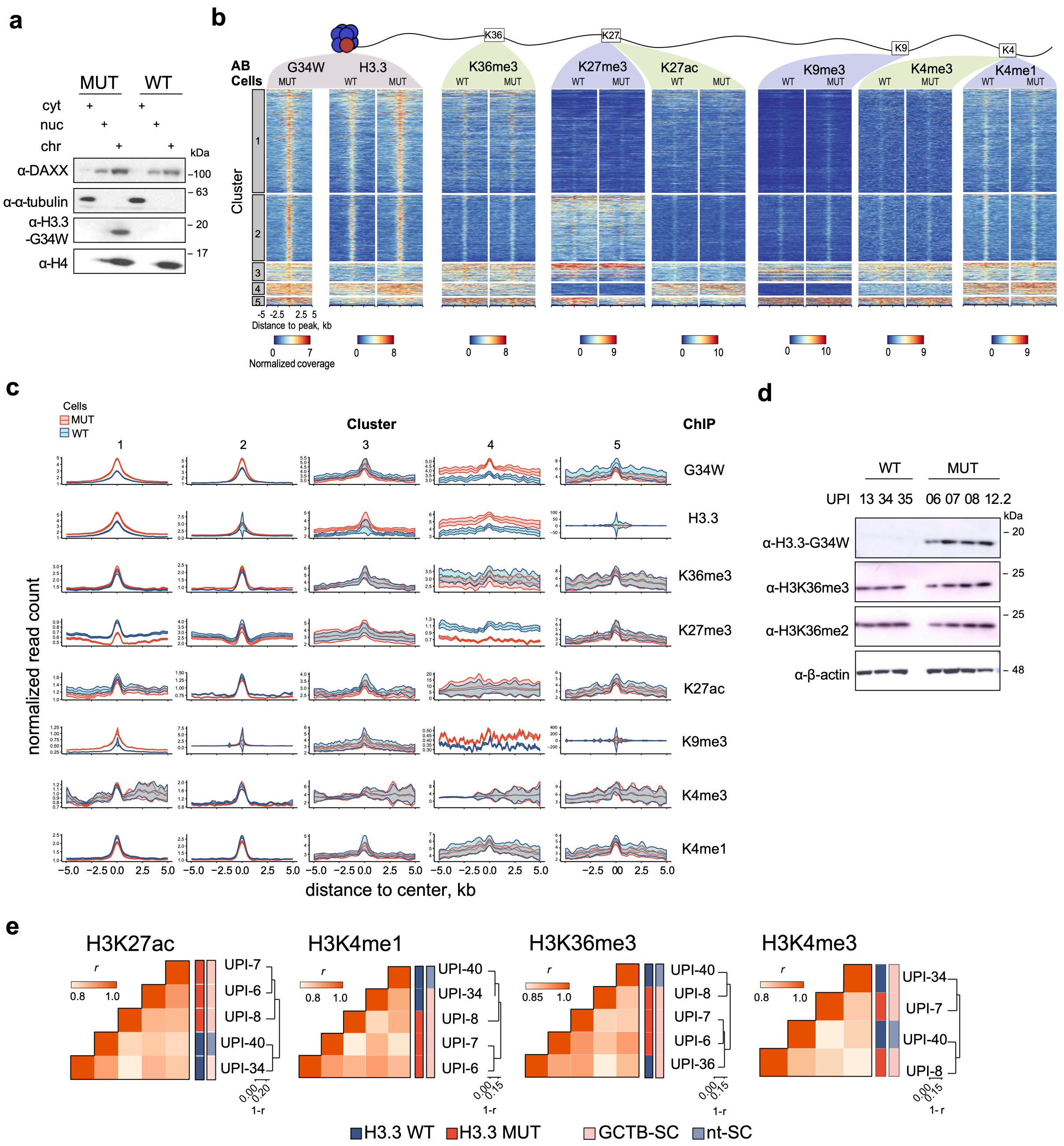

Supplementary figure 2: Genome-wide epigenetic distortion in H3.3 MUT stromal cells

a. Western blot analysis of fractionated protein from two GCTB stromal cells (unified patient identifiers, UPI-6 and UPI-13); either positive (H3.3 MUT) or negative (H3.3 WT) for H3.3-G34W. Cyt, cytosol; Nuc, nucleus; Chr, chromatin. The blot shown is representative of three independent replication experiments. **b.** Genomic summary heatmap of ChIP-seq signals of H3.3-G34W, wild type H3.3 and histone marks at H3.3-G34W incorporation regions. Each row corresponds to one of the H3.3-G34W enriched loci. Clusters were obtained using *k*-means clustering in a space spanned by the columns of the displayed heatmap. **c**. Alterations of chromatin accessibility (ATAC) and histone marks (H3K4me1, H3K4me3, H3K27ac, H3K27me3, H3K36me3 and H3K9me3) around H3.3-G34W sites. Solid lines represent the mean profiles while ribbons show standard errors of the mean across all summarized regions. Cluster numbers correspond to those given in **b**. H3.3 WT and H3.3 MUT profiles are shown in blue and red, respectively. kb, kilobases. **d**. Western blot analysis of global levels of H3K36 methylation in whole cell lysates of H3.3 WT and H3.3 MUT stromal cells. UPI-12.2 represents a relapse case. The blot shown is representative of two independent replication experiments. **e**.

Hierarchical clustering with correlation distance of H3K27ac, H3K4me1, H3K36me3 and H3K4me3 profiles in nt-SC (violet) and stromal cells (pink). H3.3 WT (blue) and H3.3 MUT (red). Heatmaps represent pairwise Pearson correlation coefficients (*r*) between respective modification profiles of two cell lines. Dendrograms were obtained with agglomerative hierarchical clustering with 1-r distance and average linkage. Heatmap color codes represent *H3F3A* mutational status (inner), and the cell type (outer), the common legend for all heatmaps is given at the bottom. GCTB-SC, GCTB stromal cells; nt-SC, nontumoral stromal cells; UPI, unified patient identifier. All analyzed stromal cells with their UPIs are listed in **Supplementary Data 1**.

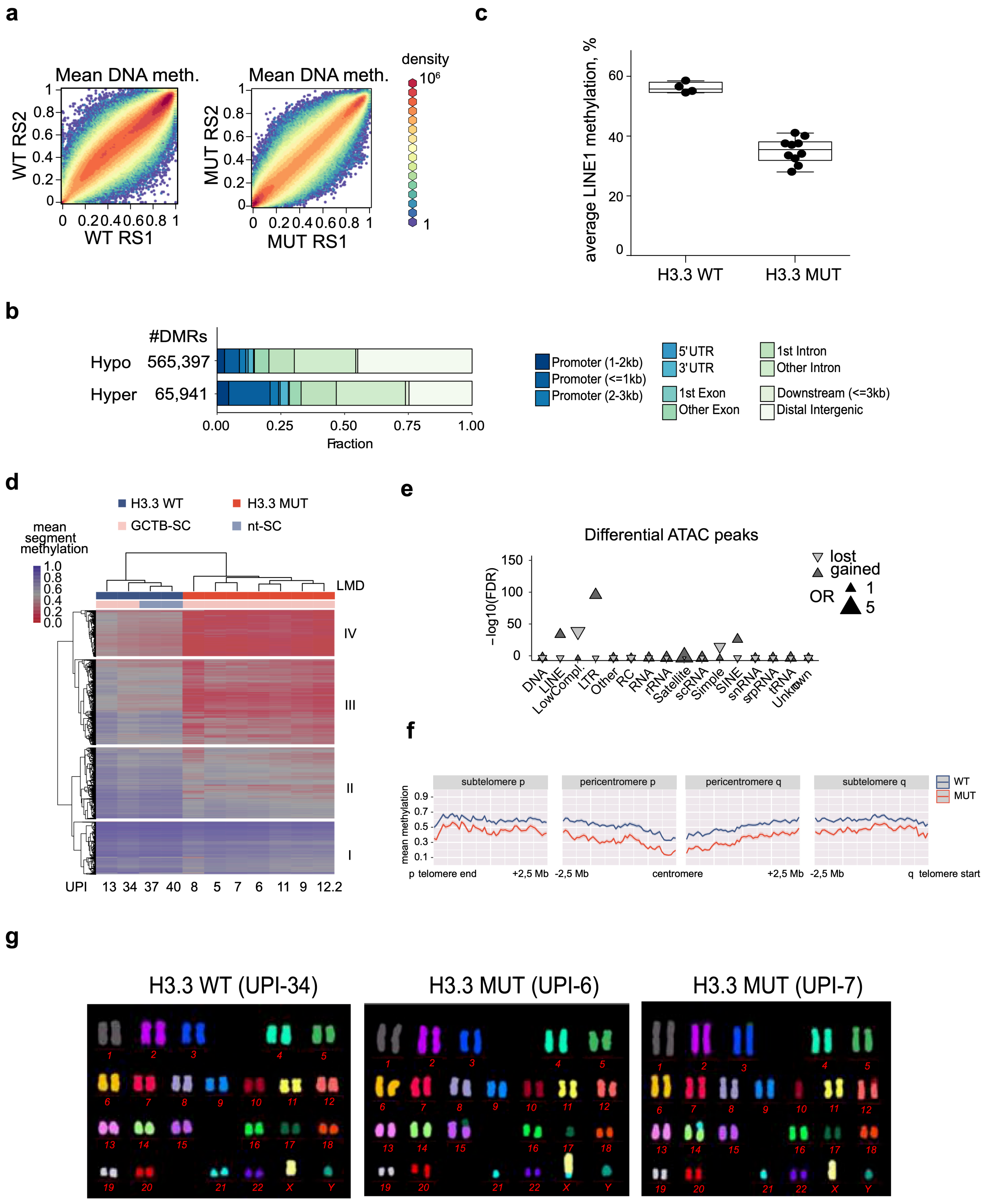

Supplementary figure 3: Heterochromatin defects in H3.3 MUT stromal cells

a. Binned scatterplot of intra-group genome wide DNA methylation profiles from WGBS in H3.3 WT cells (left) and H3.3 MUT cells (right). Each group was split into two random subsets and average profiles of the subsets were used to generate the binned scatterplots. RS1 and RS2, random sample subsets 1 and 2, respectively. Hexagon color represents binned density gradient of 1 (blue), 1000 (yellow) and 10^6 (red) points. **b**. Genomic distribution of differentially methylated regions (DMR) between H3.3 WT and H3.3 MUT cells with respect to gene model annotation. Hyper, DMRs hypermethylated in H3.3 MUT; Hypo, DMRs hypomethylated in H3.3 MUT. **c**. Quantification of average DNA methylation levels at LINE1 elements using MassARRAY in H3.3 WT and H3.3 MUT cells. Points represent individual patients (n=4 for

H3.3 WT and n=10 for H3.3 MUT), central line represents the median, box covers the inter-quartile range, whiskers extend from minimal to maximal values. Difference is significant (p=6.8·10^{-7}, unpaired two-tailed t-test). In the boxplot bar represents the mean, box the IQR, upper and lower whiskers extend from the smallest to the largest value within 1.5 IQR from the lower and upper edges of the box, respectively. **d**. Mean methylation levels of segments falling into one of the four major large methylation domains (LMD). Dendrograms over columns and rows show clustering of samples using agglomerative hierarchical clustering with Euclidean distance and average linkage. GCTB-SC, GCTB-derived stromal cells, pink; nt-SC, nontumoral stromal cells, light blue; H3.3 WT blue, H3.3 MUT red. **e.** Genomic overlap analysis of differential ATAC peaks with repetitive elements. Lost (light grey) and gained (dark grey) refer to peaks showing a decrease (disappearance) or increase (emergence) in H3.3 MUT, respectively. FDR, false discovery rate; OR, odds ratio. **f**. Mean DNA methylation profiles of subtelomeric and pericentromeric regions in H3.3 WT (blue) and H3.3 MUT cells (red). **g**. Visualization of karyotypes using m-FISH in H3.3 WT and H3.3 MUT cells.

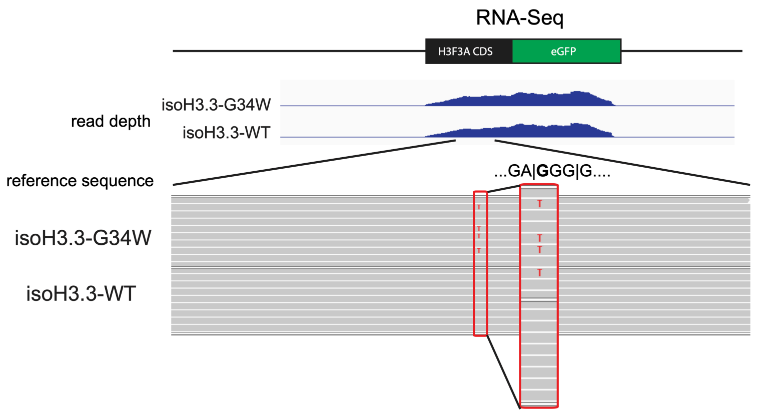

Supplementary figure 4: Recapitulation of DNA methylation alterations in an isogenic system

Genomic browser view of RNA-Seq data for *H3F3A* in iso-H3.3-G34W and iso-H3.3-WT HeLa cells showing the locus-endogenous knock-in of the G34W-mutation. IGV snapshot of the coverage depth and single-read display is made based on an alignment to the reference sequence of the full construct.

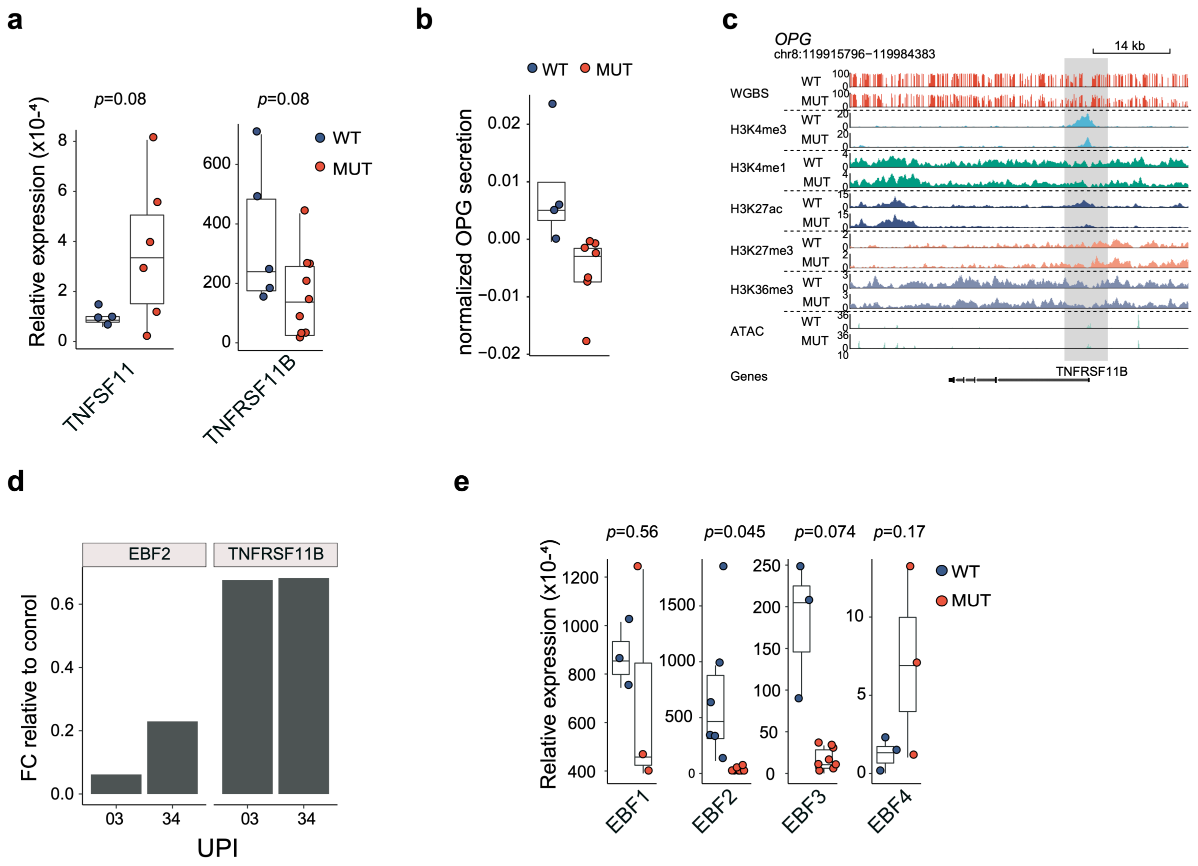

Supplementary figure 5: The epigenetic determinants of the GCTB osteolytic phenotype

a. Expression levels of RANKL (*TNFSF11*) and OPG (*TNFRSF11B*) in H3.3 WT (blue, n=4 and n=5 biologically independent cell lines for *RANKL* and *OPG*, respectively) and H3.3 MUT cells (red, n=6 and n=9 biologically independent cell lines for *RANKL* and *OPG*, respectively) analyzed with qRT-PCR**.** Expression is shown relative to *GAPDH*. An unpaired two-tailed t-test with Welch correction was used for *RANKL* and an unpaired two-tailed t-test for *OPG*. **b.** OPG ELISA with cell culture supernatant of H3.3 WT (blue) and H3.3 MUT cells (red). Amount of secreted OPG is shown normalized to cell viability as a surrogate for cell number measured by cell titer blue assay. Observations were centered per replicate and averaged for each patient. Each dot represents a different patient. An unpaired two-tailed t-test was used, *p*=0.0192, n=4 and n=7 biologically independent cell lines in the H3.3 WT and H3.3 MUT group respectively. **c**. Genomic browser view of the OPG encoding gene *TNFRSF11B*. Each lane (dash-separated) represents normalized averaged signals of DNA methylation, the level of H3K4me3, H3K4me, H3K9me3, H3K27ac, H3K27me3, H3K36me3 and ATAC in several replicates of H3.3 WT (lane-wise top) or H3.3 MUT (lane-wise bottom) cells. **d.** Expression analysis of *EBF2* and *OPG* (*TNFRSF11B*) by qRT-PCR 48 hours after siRNA mediated knockdown of *EBF2* in two H3.3 WT cell lines (UPI-03, UPI-34). Fold change (FC) relative to expression in cells transfected with a control siRNA. **e**. Expression levels of all members of the *EBF* family in H3.3 WT (blue, n=3; 6; 3 and 3 biologically independent cell lines for *EBF1*, 2, 3 and 4 respectively) and H3.3 MUT (red, n=3; 12; 8 and 3 biologically independent cell lines for *EBF1*, *2*, *3* and *4* respectively) cells analyzed with qRT-PCR**.** Expression is shown relative to *GAPDH.* An unpaired two-tailed t-test was used for *EBF1* and *EBF4*, an unpaired two-tailes t-test with Welch correction for *EBF2* and *EBF3*. In all boxplots bar represents the mean, box the IQR, upper and lower whiskers extend from the smallest to the largest value within 1.5 IQR from the lower and upper edges of the box, respectively.

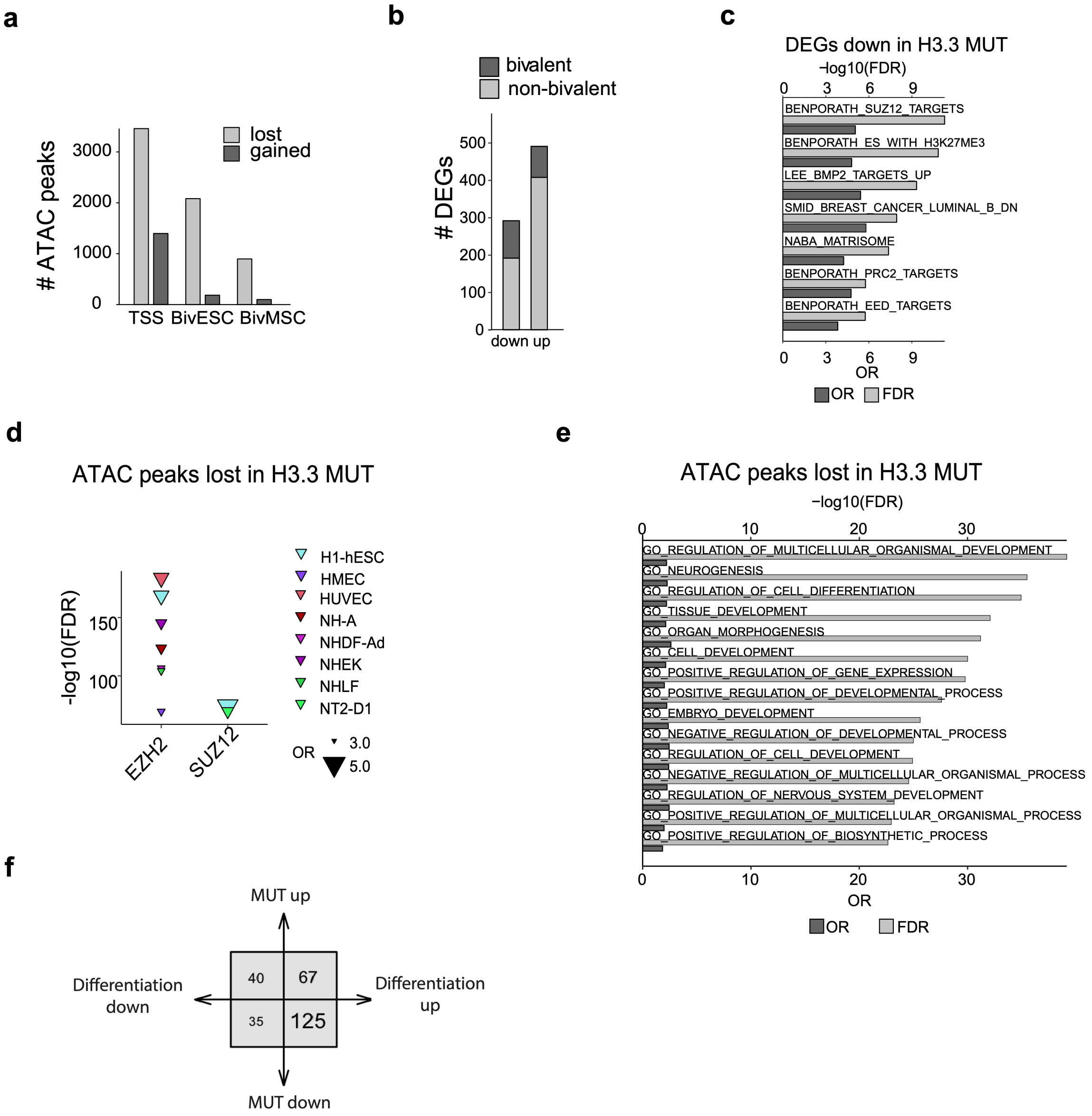

Supplementary figure 6: Delay of osteogenic differentiation in H3.3 MUT stromal cells

a. Enrichment of differentially accessible chromatin sites at transcription start sites (TSS) of bivalent genes. Bars represent numbers of differential ATAC peaks with increased (gained, dark grey) and decreased (lost, light grey) signal in H3.3 MUT cells overlapping bivalent TSSs. TSS, all Ensemble TSS; BivESC, consensus human bivalent TSS in ESC as defined in Court *et al.* (Ref. 65 in the main text); BivMSC, bivalent TSS defined in nt-SC cells using peak overlaps. **b**. Fraction of bivalent genes (dark grey) in the total set of differentially expressed genes (DEG). **c.** Top significantly enriched gene sets from the MSigDB collection of curated gene sets (C2) obtained with overrepresentation analysis of DEGs downregulated in H3.3 MUT. OR, odds ratio; FDR, false discovery rate. **d**. Genomic overlap enrichment analysis of differential ATAC peaks lost in H3.3 MUT with transcription factor binding sides (ENCODE). Symbol color labels various cell types as they appear in the *LOLA* core database. ESC, embryonic stem cell; HUVEC, human umbilical vein endothelial cells; LC, leukemia cell; OR, odds ratio. **e**. Top significantly enriched gene sets from the MSigDB collection of Gene Ontology gene sets (C5) obtained with overrepresentation analysis of genes containing one or more differential ATAC-seq peaks lost in H3.3 MUT within 10kb from a canonical TSS from Ensemble. OR, odds ratio; FDR, false discovery rate. **f**. Overlap of DEGs between H3.3 WT and H3.3 MUT stromal cells and genes significantly altered during osteogenic differentiation.

Supplementary Tables

Supplementary Table 1: Antibodies (continued on the next page)

Antibody	Manufacturer	Cat. #	Species reactivity	Applications
monoclonal mouse anti-β actin C4	Santa Cruz, Santa Cruz, USA	sc-47778	mouse, rat, human, avian, bovine, canine, porcine, rabbit, dictyostelium discoideum, physarum polycephalum	WB, IP, IF, IHC, ELISA
monoclonal mouse anti-β actin C4 HRP	Santa Cruz, Santa Cruz, USA	sc-47778 HRP	mouse, rat, human, avian, bovine, canine, porcine, rabbit, dictyostelium discoideum, physarum polycephalum origin	WB, IP, IF, IHC, ELISA
polyclonal rabbit anti-Histone H3.3	Merck Millipore, Burlington, Massachusetts, USA	09-838	mouse, human	WB, ICC, DB, ChIP
monoclonal rabbit anti-Histone H3.3	Abcam, Cambridge, UK	ab176840	mouse, rat, human,	ChIP, DB, ICC, IHC, WB
monoclonal rabbit anti-H3.3-G34W	RevMab Bioscience, San Francisco, USA	31-1145-00		WB, ELISA, IHC, ICC, ChIP
rabbit anti-H3.3-G34W	Active motif, Carlsbad, USA	61805	not commercially available yet	
polyclonal rabbit anti-H3K27me3	Merck Millipore, Burlington, Massachusetts, USA	07-449	mouse, human	ICC, IP, WB, IHC
polyclonal rabbit anti-H3K36me3	Abcam, Cambridge, UK	ab9050	mouse, rat, cow, human, saccharomyces cerevisiae, xenopus laevis, arabidopsis thaliana, caenorhabditis elegans, drosophila melanogaster, schizosaccharomyces pombe, zebrafish, Silk worm, rice, xenopus tropicalis, trypanosoma brucei	ICC/IF, WB, ChIP
monoclonal rabbit anti-H3K36me2	Cell Signaling Technology, Danvers, USA	2901	human, mouse, rat, monkey	WB, ICH, ICC, FC
polyclonal rabbit anti-Histone H4	Merck Millipore, Burlington, Massachusetts, USA	07-108	human, mouse, bovine, chicken, xenopus	WB
polyclonal rabbit anti-H3	Abcam, Cambridge, UK	ab1791	mouse, rat, chicken, dog, human, saccharomyces cerevisiae, xenopus laevis, arabidopsis thaliana, caenorhabditis elegans, drosophila melanogaster, ferret, indian muntjac, schizosaccharomyces pombe, zebrafish, silk worm, dictyostelium discoideum, rainbow trout, trypanosoma cruzi, neurospora crassa, toxoplasma gondii, rice, schistosoma mansoni, cyanidioschyzon merolae	IHC, M, ChIP, IP, WB

Antibody	Manufacturer	Cat. #	Species reactivity	Applications
polyclonal rabbit anti-H3K4me1	Abcam, Cambridge, UK	ab8895	mouse, human, pig, saccharomyces cerevisiae, tetrahymena, xenopus laevis, drosophila melanogaster, plasmodium falciparum, xenopus tropicalis	ICC/IF, ChIP, WB, IHC-P
polyclonal rabbit anti-H3K4me3	Active motif, Carlsbad, USA	39915	budding yeast, human wide range predicted	ChIP, ICC/IF, WB
polyclonal rabbit anti-H3K27ac	Active motif, Carlsbad, USA	39133	budding yeast, human wide range predicted	ChIP, ICC/IF, WB
polyclonal rabbit anti-H3K9me3	Diagenode, Denville, USA	C15410056	thale cress, zebrafish, human, mouse	ChIP, DB, ELISA, IF, WB
polyclonal rabbit anti-H3K27me3	Diagenode, Denville, USA	C15410069	human, mouse, rat, pig, zebrafish, drosophila, schistosoma, arabidopsis, cow	ChIP, ELISA, DB, WB, IF
monoclonal mouse anti-α-tubulin	Sigma Aldrich, St. Louis, USA	T6199	bovine, rat, yeast, human, mouse, chicken, fungi, amphibian	IHC, IP, WB
polyclonal rabbit anti-DAXX	Santa Cruz, Santa Cruz, USA	sc-7152	human, mouse, rat	WB, IF,ChIP, ICC
monoclonal mouse anti-CD45-APC	Invitrogen, Carlsbad, USA	17-0459-42	human	FC
monoclonal mouse anti-CD235-APC	Invitrogen, Carlsbad, USA	17-9987-42	human	FC
monoclonal mouse anti-CD105-FITC	Biolegend, San Diego, USA	323204	human	FC
monoclonal mouse anti-CD90-APC-Cy7	Biolegend, San Diego, USA	328132	human, african green, baboon, cynomolgus, pigtailed macaque, rhesus, swine	FC
monoclonal mouse anti-FLAG	Sigma Aldrich, St. Louis, USA	F3165	epitope tag/fusion protein	WB
goat anti-avidin	Vector, Burlingame, USA	BA-0300		IF
polyclonal rabbit anti-digoxin	Sigma Aldrich, St. Louis, USA	D7782		FISH, ChIP,
polyclonal goat anti-rabbit Cy5.5	Linaris, Mannheim, Germany	PAK0027	rabbit	IF, WB
streptavidin Alexa Fluor 750 conjugate	Invitrogen	S21384		IF

WB, Western Blotting; IP, Immunoprecipitation; IF, Immunofluorescence; IHC, Immunohistochemistry; ELISA, Enzyme linked immunosorbent assay; ICC, Immunocytochemistry; DB, Dot Blot; ChIP, Chromatin immunoprecipiatation; M-microscopy; FC, Flow Cytometry;e FISH, fluorescence in situ hybridization.

Supplementary Table 2: Commercially available cell lines

Cell line	Commercial source	Authentication method	Authentication date
Hos143B	Sigma-Aldrich	COA DSMZ; full matching STR reference profile.	09/2018
CAL-72	DSMZ	COA Multiplexion; SNP-based assay 100%	09/2018
HEK293T	DSMZ	COA Multiplexion; SNP-based assay 100%	06/2020
HeLa	KCLB	COA KCLB; full matching STR reference profile.	03/2018
