# Supplementary material for: Globally altered epigenetic landscape and delayed osteogenic differentiation in H3.3-G34W-mutant giant cell tumor of bone
Source: Nat Commun. 2020 Oct 27;11:5414. doi: 10.1038/s41467-020-18955-y (PMC7591516; doi:10.1038/s41467-020-18955-y)
Supplement: Supplementary file 2 — Description of Additional Supplementary Files [file 41467_2020_18955_MOESM2_ESM.pdf]

## Description of Additional Supplementary Files

**Supplementary data 1: Sample annotation and omics data information**.

Sheet Samples lists all patients and biological specimens used in the study. Note that in some cases several different sample types from one patient (tissue, stromal cells, blood) were used, as specified in the column Material. Column Source specifies the cohort: UHOK, Heidelberg; KCC, Seoul; UKHE, Hamburg; UKL, Leipzig. Column H3.3-G34W IHC gives the result of immunohistochemical detection of the mutant histone with anti-H3.3-G34W antibody. Column H3F3A_status (msPCR) and H3F3A_status (Sanger) give the results of *H3F3A* mutation status screening using mutation-specific PCR and sequencing respectively. Some of the tumours were subsequently reclassified by a pathologist (given in the Comments column).

Sheets WGS, 450k, WGBS, ATAC-Seq, ChIP-mentation, RNA-Seq and H3F3A MiSeq provide information about samples used for each omics assay, with corresponding QC information.

**Supplementary data 2: H3.3-G34W enrichment regions and large methylation domains**.

Sheet LMDs contains genomic coordinates (columns chr, start, end), width and type (column LMD) of identified large methylation domains as well as their average methylation in H3.3 WT and MUT cells (columns avgMeth_WT and avgMeth_MUT, respectively).

Sheets H3.3 regions WT, H3.3 regions MUT and H3.3-G34W regions contains genomic coordinates (columns chr, start, end) and width of identified H3.3 enrichment regions in H3.3WT and H3.3 MUT cells and H3.3-G34W enrichment regions in H3.3 MUT cells, respectively.

All coordinates are for GRC 37 (hg19) assembly of the human genome.

**Supplementary data 3: Differentially accessible regions, functional annotation and motif enrichment analysis.**

Differentially accessible regions are listed in the sheet ATAC diff. peaks. Columns Chromosome, Start, End, Width give genomic coordinates and width of each region. Log-fold-change of attach signal is given in the column logFC, whereas columna PValue gives differential read count p-value from edgeR. Column Class classifies each peak into lost (logFC<0) and gained (logFC>0) as it appears throughout the manuscript. Columns TSS_RefSeq, TSS_Biv_ESC and TSS_Biv_MSC contain TRUE for regions that overlap any RefSeq TSS, an ESC-specific bivalent TSS or an MSC-specific bivalent TSS, respectively

Subsequent sheets contain the results of functional annotation and motif enrichment analyses. In the sheet names: ATAC lost, differentially accessible regions with decreased accessibility in H3.3 MUT cells; ATAC gained, differentially accessible regions with increased accessibility in H3.3 MUT cells.

Results of functional annotation of differentially accessible regions were performed with LOLA package and are given in the sheets with the names starting with “LOLA” token. Two databases, the official LOLA Core and a custom RepeatMasker-based one were used for the analysis, specified as the last token in names of corresponding sheets.

The LOLA sheet columns are described in the documentation of the runLOLA() function: userSet and dbSet: index into their respective input region sets. pvalueLog: -log10(pvalue) from the Fisher's exact test result; oddsRatio: odds ratio from the Fisher's exact test; support: number of regions in userSet overlapping databaseSet; rnkPV, rnkOR, rnkSup: rank based on p-value, odds-ratio, and support respectively. maxRnk, meanRnk: max and mean of the 3 previous ranks, providing a combined ranking system. b, c, d: 3 other values completing the 2x2 contingency table (with support). qValue: q value after the Benjamini-Hochberg adjustment, size: total number of regions in the region set. The remaining columns describe the dbSet for the row.

Results of the Motif enrichment analysis with HOMER tool are given in the sheets with the names starting with “HOMER” token. Column Motif Name and Consensus provide common name and consensus sequence of each motif. P-value, log P-value and q-value provide original, -log10-transformed and Benjamini-Hochberg adjusted P-values of enrichment analysis, respectively. Further columns provide numbers and percentages of regions containing the motif in the target and background sets.

**Supplementary data 4: Differentially expressed genes, term overrepresentation and gene-set enrichment analysis.**

Identified significantly down- and upregulated differentially expressed genes are listed in sheets DEGs, down and DEGs, up, respectively. In both sheets columns A to J are part of the standard DESeq2 output: ENSEMBLE_ID, gene identifier in the Ensemble transcript database; Symbol, official gene symbol, in case available; Entrez_ID, gene identifier in the Entrez database; baseMean, the average of the normalized count values, dividing by size factors, taken over all samples; log2FoldChange, estimate of the effect size on log2 scale, lfcSE, the standard error estimate for the log2 fold change estimate; TestStatistic, the value of the test statistic for the gene or transcript; Pvalue, P value of the test for the gene or transcript; Padj, P value after adjusting for multiple testing. Columns Biv_hESC and Bic_MSC have TRUE for genes that were identified as bivalent in hESCs or MSCs, respectively. PRC2_target column specifies whether a gene was identified as a target of Polycomb repressive complex in human embryonic fibroblasts according to Bracken et al. (reference 82 of the main manuscript).

Sheet ORA lists identifiers of terms and gene sets from MSigDB database (v.6.2) significantly overrepresented in down- and upregulated DEGs. Column DEG set, specifies the result set for which overrepresentation was found, either down- or upregulated genes. Column MSigDB Collection gives the gene set collection to which the overrepresented term belongs (see comprehensive description of the collections at https://www.gsea-msigdb.org/gsea/msigdb/collections.jsp).

Columns GeneSet gives the ID of the overrepresented term or gene set. Columns OR, Pvalue and FDR specify odds-ratio, P value and false discovery rate of the found overrepresentation, respectively

Sheet GSEA gives results of the gene set enrichment analysis with R package *fgsea*. Columns MSigDB Collection and GeneSet same as in sheet ORA above. Columns ES, FDR, -log10(FDR) give the absolute score value, false discovery rate and its negative log10 value of for each enrichment, respectively. Column Direction specifies the sign of the enrichment score, positive for the upregulated sets and negative for the downregulated ones.

Sheet Overlap DEGs, MSC. Diff. GCTB lists all differentially expressed genes that overlapped between GCTB MUT vs WT analysis and in the analysis of MSC differentiation dataset.

Column Class gives the category of the overlap as defined in Supplementary Figure 5f. Columns Gene and Symbol provide Ensemble ID and official gene symbol for each overlapping gene. Subsequent columns contain results of the differential expression analysis using DESeq2, with prefixes DIFF and GCTB assigning the results to MSC differentiation and GCTB experiment, respectively; baseMean: mean expression of the gene across all samples; log2FoldChange, lfsSE: standard log2-fold change (LFC) from a two-group comparison and its standard error; log2FoldChange_LRT, DIFF_lfcSE_LRT: LFC of the likelihood ratio test for association with the differentiation time variable and its standard error; log2FoldChange_504_to_0: for MSC differentiation, LFC between the last and zero time-points; stat, Pvalue, Padj: DESeq2 test statistic, the original and multiple-testing adjusted P-values, respectively.

**Supplementary data 5: Oligonucleotide sequences**.

Sheet H3F3A mutation lists primers used for various assays for detecting H3F3A mutations in patient samples, including mutation-specific PCR, Sanger sequencing and deep resequencing using MiSeq platform.

Sheet qRT-PCR lists primers used for gene expression measurements with quantitative reverse transcription PCR.

Sheet MassARRAY lists primers used to perform methylation measurements using the SEQUENOM MassARRAY platform.

Sheet WGBS sequencing lists customized Illumina P5 and P7 sequencing primers.
