# Supplementary material for: Globally altered epigenetic landscape and delayed osteogenic differentiation in H3.3-G34W-mutant giant cell tumor of bone
Source: Nat Commun. 2020 Oct 27;11:5414. doi: 10.1038/s41467-020-18955-y (PMC7591516; doi:10.1038/s41467-020-18955-y)
Supplement: Supplementary file 8 — Reporting Summary [file 41467_2020_18955_MOESM8_ESM.pdf]

nature research

Corresponding author(s): Christoph Plass, Anders Lindroth

Last updated by author(s): Aug 25, 2020

Reporting Summary

Nature Research wishes to improve the reproducibility of the work that we publish. This form provides structure for consistency and transparency in reporting. For further information on Nature Research policies, see Authors & Referees and the Editorial Policy Checklist.

Statistics

For all statistical analyses, confirm that the following items are present in the figure legend, table legend, main text, or Methods section.

n/a	Confirmed	
☐	☒	The exact sample size (*n*) for each experimental group/condition, given as a discrete number and unit of measurement
☐	☒	A statement on whether measurements were taken from distinct samples or whether the same sample was measured repeatedly
☐	☒	The statistical test(s) used AND whether they are one- or two-sided<br>*Only common tests should be described solely by name; describe more complex techniques in the Methods section.*
☐	☒	A description of all covariates tested
☐	☒	A description of any assumptions or corrections, such as tests of normality and adjustment for multiple comparisons
☐	☒	A full description of the statistical parameters including central tendency (e.g. means) or other basic estimates (e.g. regression coefficient) AND variation (e.g. standard deviation) or associated estimates of uncertainty (e.g. confidence intervals)
☐	☒	For null hypothesis testing, the test statistic (e.g. *F*, *t*, *r*) with confidence intervals, effect sizes, degrees of freedom and *P* value noted<br>*Give P values as exact values whenever suitable.*
☒	☐	For Bayesian analysis, information on the choice of priors and Markov chain Monte Carlo settings
☒	☐	For hierarchical and complex designs, identification of the appropriate level for tests and full reporting of outcomes
☐	☒	Estimates of effect sizes (e.g. Cohen's *d*, Pearson's *r*), indicating how they were calculated

Our web collection on statistics for biologists contains articles on many of the points above.

Software and code

Policy information about availability of computer code

Data collection

Collection of sequencing data was performed with standard Illumina software. Western blots were imaged on a Amersham Imager 680 (GE, USA) machine. qRT-PCRs were quantified on LightCycler 480 (Roche, Germany), MassARRAY quantification was performed with Sequenom (Agena Bioscience, USA).

Data analysis

Most of the data processing and analytical steps were performed using established software tools: bwa mem (v. 0.7.8), Bowtie2 (v. 2.3.0), cutadapt (v. 1.10), Trimmomatic (v. 0.36), Picard (v. 1.125), biobambam (v.0.0.148), Platypus (v. 1.0), ANNOVAR (v. 2017Jul16), SOPHIA (v.34.0), ACEseq (v. 1.0), deepTools (v. 2.3.3) , Macs2 (v. 2.1.1.), ChIPpeakAnno (v. 3.18.0), HOMER (v. 4.9), ChromHMM (v. 1.18), MethylDackel (v. 0.3.0), BSmooth (v. 1.4.0), DSS (v. 2.27.0), RnBeads (v. 2.2.0), HISAT2 (v. 2.0.4), StringTie (v. 1.3.3), edgeR (v. 0.3.16), DeSeq2 (v. 1.18.1) , changepoint (v. 2.2.2), LOLA (v. 1.8.0), GeneOverlap (v. 1.14.0), fgsea (v. 1.4.1).

DKFZ/PCAWG WGS analysis workflow is available as a Docker container from:
https://dockstore.org/containers/quay.io/pancancer/pcawg-dkfz-workflow

Compatible CWL workflows for WGBS, ATAC-Seq and ChIP-Seq data processling are publicly available on Github/CompEpigen:
https://github.com/CompEpigen/WGBS_workflows (BWA_meth_start_with_trimmed.cwl)
https://github.com/CompEpigen/ATACseq_workflows
https://github.com/CompEpigen/ChIPseq_workflows
A snakemake-based processing workflow for RNA-seq data is available at https://github.com/CompEpigen/RNASeq_GCTB.

Custom code for LMD calling using changepoint algorithms is available at https://github.com/lutsik/CP-PMD

Other custom code is available at https://github/lutsik/GCTB_Epigenome.

For manuscripts utilizing custom algorithms or software that are central to the research but not yet described in published literature, software must be made available to editors/reviewers. We strongly encourage code deposition in a community repository (e.g. GitHub). See the Nature Research guidelines for submitting code & software for further information.

Data

Policy information about availability of data

All manuscripts must include a data availability statement. This statement should provide the following information, where applicable:

- Accession codes, unique identifiers, or web links for publicly available datasets
- A list of figures that have associated raw data
- A description of any restrictions on data availability

All raw patient-derived sequencing data from WGS, WGBS, ATAC-seq, ChIP-seq, RNA-seq and deep targeted resequencing have been deposited in the European Genome-Phenome Archive (EGA) under restricted access with the accession code: EGAS00001003730. Processed sequencing data and microarray data have been deposited in ArrayExpress with the accession code: E-MTAB-7184. RNA-Seq data of the MSC osteogenic differentiation experiment are deposited at the Gene Expression Omnibus (GEO) with the accession code: GSE129036.

We furthermore used gene sets from the Molecular Signatures Database v. 6.2 (MSigDB):
https://www.gsea-msigdb.org/gsea/msigdb/download_file.jsp?filePath=/msigdb/release/6.2/msigdb_v6.2_files_to_download_locally.zip ,
ChromHMM states of human bone-marrow by ENCODE project (sample E026):
https://egg2.wustl.edu/roadmap/data/byFileType/chromhmmSegmentations/ChmmModels/coreMarks/jointModel/final/E026_15_coreMarks_segments.bed ,
Replication timing segments by Repliscan project (Hansen et al, 2010):
https://de.cyverse.org/anon-files/iplant/home/gzynda/public/hansen2010_replicate/repliscan_50kb.gff3 ,
Consensus list of ESC-specific bivalent genes (Court and Arnaud, 2017):
http://www.oncotarget.com/index.php?journal=oncotarget&page=article&op=downloadSuppFile&path%5B%5D=13746&path%5B%5D=21048
LOLA Core database of functionally annotated genomic regions:
http://big.databio.org/regiondb/LOLACore_180423.tgz
Database of common variant calls from the 1000 Genomes Project:
http://ftp.1000genomes.ebi.ac.uk/vol1/ftp/release/20130502/
GRCh37 transcript database
ftp://ftp.ncbi.nlm.nih.gov/refseq/H_sapiens/annotation/GRCh37_latest/refseq_identifiers/GRCh37_latest_genomic.gff.gz
Repeat masker database:
http://www.repeatmasker.org

Field-specific reporting

Please select the one below that is the best fit for your research. If you are not sure, read the appropriate sections before making your selection.

☒ Life sciences ☐ Behavioural & social sciences ☐ Ecological, evolutionary & environmental sciences

For a reference copy of the document with all sections, see nature.com/documents/nr-reporting-summary-flat.pdf

Life sciences study design

All studies must disclose on these points even when the disclosure is negative.

Sample size	Our study is based on a cohort of 96 GCTB patients and 8 non-tumor samples. Individual data layers were obtained by sub-sampling this cohort with numbers specified for individual analyses and stated in supplementary material (Supplementary Data file 1). No sample-size calculations were carried out and each omics analysis was performed on the maximal number of primary cell lines given the availability of patient material and a limited number of passages attainable for the primary cells in culture.
Data exclusions	Data was excluded based on QC results for individual assays. Two samples were excluded from RNA-Seq analysis based on low values for the RNA integrity number, a QC criterion predefined by the DKFZ Genomics and Proteomics Core Facility. One WGBS sample was excluded after the initial exploratory analysis as a strong outlier in PCA
Replication	NGS-based experiments (WGS, WGBS, ATAC-Seq, ChIP-mentation, RNA-Seq) were conducted as single experiments with sample sizes specified in Supplementary Data file 1. No independent replication experiments were attempted.<br>All comparisons in Western blots, MASSArray and qRT-PCR is based on two or more patients or independent isogenic cell lines. Analysis of isogenic H3.3 knock-in was performed in 4 independent HeLa clones, without subsequent replication. EBF2 knock-down was performed in 4 replicates in one patient cell line. Results were successfully replicated in two additional independent patient cell lines (one replicate each).<br>qRT-PCRs were performed with at least three technical replicates.
Randomization	Not applicable since the samples are derived from fixed experimental groups.
Blinding	Not applicable for most analyses due to sensible growth differences between H3.3 WT and H3.3 MUT cells, obvious to the experimenter.

Reporting for specific materials, systems and methods

We require information from authors about some types of materials, experimental systems and methods used in many studies. Here, indicate whether each material, system or method listed is relevant to your study. If you are not sure if a list item applies to your research, read the appropriate section before selecting a response.

Materials & experimental systems

n/a	Involved in the study
☐	☒ Antibodies
☐	☒ Eukaryotic cell lines
☒	☐ Palaeontology
☒	☐ Animals and other organisms
☐	☒ Human research participants
☒	☐ Clinical data

Methods

n/a	Involved in the study
☐	☒ ChIP-seq
☒	☐ Flow cytometry
☒	☐ MRI-based neuroimaging

Antibodies

Antibodies used

Information about all antibodies used in the study, including supplier, catalog number, species reactivity and intended applications are given in Supplementary Table 1. All dilutions are specified in Methods section where appropriate.

Validation

For the validation of the H3.3-G34W antibody a Western blot was performed with cells containing the mutated histone. Cross-reactivity of the H3.3-G34W antibody was additionally verified with a Histone code peptide array (JPT, Berlin, Germany) containing over 3000 histone peptides (data available upon request). Statement also included into the "ChIP-sequencing and analysis" subsection of the Methods section.

For all other antibodies we relied upon suppliers' validation for species reactivity and applications. Respective statements from supplier websites are given in Supplementary Table 1 and below

Antibody;Manufacturer;Cat. #;Species reactivity;Applications

monoclonal mouse anti-actin C4 ;Santa Cruz, Santa Cruz, USA;sc-47778;mouse, rat, human, avian, bovine, canine, porcine, rabbit, dictyostelium discoideum, physarum polycephalum;WB, IP, IF, IHC, ELISA
monoclonal mouse anti-actin C4 HRP;Santa Cruz, Santa Cruz, USA;sc-47778 HRP;mouse, rat, human, avian, bovine, canine, porcine, rabbit, dictyostelium discoideum, physarum polycephalum origin;WB, IP, IF, IHC, ELISA
polyclonal rabbit anti-Histone H3.3 ;Merck Millipore, Burlington, Massachusetts, USA;09-838;mouse, human;WB, ICC, DB, ChIP
monoclonal rabbit anti-Histone H3.3;Abcam, Cambridge, UK;ab176840;mouse, rat, human,;ChIP, DB, ICC, IHC, WB
monoclonal rabbit anti-H3.3-G34W;RevMab Bioscience, San Francisco, USA;31-1145-00;;WB, ELISA, IHC, ICC, ChIP
rabbit anti-H3.3-G34W;Active motif, Carlsbad, USA;61805;not commercially available yet;
polyclonal rabbit anti-H3K27me3;Merck Millipore, Burlington, Massachusetts, USA;07-449;mouse, human;ICC, IP, WB, IHC
polyclonal rabbit anti-H3K36me3;Abcam, Cambridge, UK;ab9050;mouse, rat, cow, human, saccharomyces cerevisiae, xenopus laevis, arabidopsis thaliana, caenorhabditis elegans, drosophila melanogaster, schizosaccharomyces pombe, zebrafish, Silk worm, rice, xenopus tropicalis, trypanosoma brucei;ICC/IF, WB, ChIP
monoclonal rabbit anti-H3K36me2;Cell Signaling Technology, Danvers, USA;2901;human, mouse, rat, monkey;WB, ICH, ICC, FC
polyclonal rabbit anti-Histone H4;Merck Millipore, Burlington, Massachusetts, USA;07-108;human, mouse, bovine, chicken, xenopus;WB
polyclonal rabbit anti-H3;Abcam, Cambridge, UK;ab1791;mouse, rat, chicken, dog, human, saccharomyces cerevisiae, xenopus laevis, arabidopsis thaliana, caenorhabditis elegans, drosophila melanogaster, ferret, indian muntjac, schizosaccharomyces pombe, zebrafish, silk worm, dictyostelium discoideum, rainbow trout, trypanosoma cruzi, neurospora crassa, toxoplasma gondii, rice, schistosoma mansoni, cyanidioschyzon merolae;IHC, M, ChIP, IP, WB
polyclonal rabbit anti-H3K4me1;Abcam, Cambridge, UK;ab8895;mouse, human, pig, saccharomyces cerevisiae, tetrahymena, xenopus laevis, drosophila melanogaster, plasmodium falciparum, xenopus tropicalis;ICC/IF, ChIP, WB, IHC-P
polyclonal rabbit anti-H3K4me3;Active motif, Carlsbad, USA;39915;budding yeast, human wide range predicted; ChIP, ICC/IF, WB
polyclonal rabbit anti-H3K27ac;Active motif, Carlsbad, USA;39133;budding yeast, human wide range predicted;ChIP, ICC/IF, WB
polyclonal rabbit anti-H3K9me3;Diagenode, Denville, USA;C15410056;thale cress, zebrafish, human, mouse;ChIP, DB, ELISA, IF, WB
polyclonal rabbit anti-H3K27me3;Diagenode, Denville, USA;C15410069;human, mouse, rat, pig, zebrafish, drosophila, schistosoma, arabidopsis, cow;ChIP, ELISA, DB, WB, IF
monoclonal mouse anti-_-tubulin;Sigma Aldrich, St. Louis, USA;T6199;bovine, rat, yeast, human, mouse, chicken, fungi, amphibian;IHC, IP, WB
polyclonal rabbit anti-DAXX;Santa Cruz, Santa Cruz, USA;sc-7152;human, mouse, rat;WB, IF,ChIP, ICC
monoclonal mouse anti-CD45-APC;Invitrogen, Carlsbad, USA;17-0459-42;human;FC
monoclonal mouse anti-CD235-APC;Invitrogen, Carlsbad, USA;17-9987-42;human;FC
monoclonal mouse anti-CD105-FITC;Biolegend, San Diego, USA;323204;human;FC
monoclonal mouse anti-CD90-APC-Cy7;Biolegend, San Diego, USA;328132;human, african green, baboon, cynomolgus, pigtailed macaque, rhesus, swine;FC
monoclonal mouse anti-FLAG;Sigma Aldrich, St. Louis, USA;F3165;epitope tag/fusion protein;WB
goat anti-avidin;Vector, Burlingame, USA;BA-0300;;IF
polyclonal rabbit anti-digoxin;Sigma Aldrich, St. Louis, USA;D7782;;FISH, ChIP,
polyclonal goat anti-rabbit Cy5.5;Linaris, Mannheim, Germany;PAK0027;rabbit;IF, WB
streptavidin Alexa Fluor 750 conjugate;Invitrogen;S21384;;IF

Eukaryotic cell lines

Policy information about cell lines

Cell line source(s)	We used 4 commercially available cell lines in this study:<br><br>Hos143B(Sigma-Aldrich)<br>CAL-72 (DSMZ)<br>HEK293T (DSMZ)<br>HeLa (Korean Cell Line Bank)
Authentication	All commercially available cell lines used within this work were authenticated: Hos143B: DSMZ 09/2018, full matching STR reference profile; CAL-72: COA Multiplexion 09/2018; identity based on a SNP-based assay 100%; HEK293T: COA Multiplexion 06/2020, identity based on a SNP-based assay 100%; HeLa: Korean Cell Line Bank, COA 03/2018, full matching STR reference profile.
Mycoplasma contamination	Patient and isogenic cell lines were verified with Venoer GEM Classic mycoplasm PCR detection kit on a regularly basis. The batches where contamination was detected were excluded from further analysis.
Commonly misidentified lines<br>(See ICLAC register)	None used.

Human research participants

Policy information about studies involving human research participants

Population characteristics	The study involved samples of GCTB patients admitted in Germany (Heidelberg, Erlangen, Hamburg) and Republic of Korea (Seoul). 56% were male, 73% were of Caucasian and 27% of Asian origin, ages ranging from 22 to 63 y.o.
Recruitment	Recruitment of patients was based on regular admission of cancer patients for the management of giant cell tumors of bone. More details are available in the Methods. To the best of our knowledge there were no sources of (self-)selection bias.
Ethics oversight	The use of patient samples and the experiments performed in this study was approved by and in accordance with guidelines and regulations by the Ethics Committees of the University of Heidelberg, University Clinic of Leipzig, University Medical Center Hamburg-Eppendorf, and the National Cancer Center of Korea (IRB NCC2015-0070).

Note that full information on the approval of the study protocol must also be provided in the manuscript.

ChIP-seq

Data deposition

☒ Confirm that both raw and final processed data have been deposited in a public database such as GEO.

☒ Confirm that you have deposited or provided access to graph files (e.g. BED files) for the called peaks.

Data access links<br>*May remain private before publication.*	Due to the access restrictions (patient data) raw adata from all experiments was being deposited at the European Genome-Phenome Archive (EGA), accession EGAS00001003730. Processed data are available from ArrayExpress, accession codes E-MTAB-7184 (ChIP-Seq), E-MTAB-9512 (ATAC-Seq), E-MTAB-9513 (WGBS), E-MTAB-9515 (RNA-Seq).
Files in database submission	UPI-13-C_K9me3.bw<br>UPI-3-C_G34W.bw<br>UPI-3-C_H3.bw<br>UPI-3-C_H3.3.bw<br>UPI-3-C_K27me3.bw<br>UPI-34-C_H3.bw<br>UPI-34-C_K27ac.bw<br>UPI-34-C_K27me3.bw<br>UPI-34-C_K36me3.bw<br>UPI-34-C_K4me1.bw<br>UPI-34-C_K4me3.bw<br>UPI-40-C_H3.bw<br>UPI-40-C_K27ac.bw<br>UPI-40-C_K27me3.bw<br>UPI-40-C_K36me3.bw<br>UPI-40-C_K4me1.bw<br>UPI-40-C_K4me3.bw<br>UPI-6-C_H3.bw<br>UPI-6-C_H3.3.bw<br>UPI-6-C_G34W.bw<br>UPI-6-C_K27ac.bw<br>UPI-6-C_K27me3.bw<br>UPI-6-C_K36me3.bw

UPI-6-C_K4me1.bw
UPI-7-C_H3.bw
UPI-7-C_H3.3.bw
UPI-7-C_G34W.bw
UPI-7-C_K27ac.bw
UPI-7-C_K27me3.bw
UPI-7-C_K36me3.bw
UPI-7-C_K4me1.bw
UPI-7-C_K4me3.bw
UPI-7-C_K9me3.bw
UPI-8-C_H3.bw
UPI-8-C_K27ac.bw
UPI-8-C_K27me3.bw
UPI-8-C_K36me3.bw
UPI-8-C_K4me1.bw
UPI-8-C_K4me3.bw

Genome browser session (e.g. UCSC)	All used peaks are available as an archive from: https://de.cyverse.org/dl/d/C0A2147D-2AF9-47F4-9BC0-32C577747E59/lutsik_etal_chipseq_peaks.tar.gz

Methodology

Replicates	List of replicates is given in Supplementary Data 1.
Sequencing depth	All sequencing depth data is given in Supplementary Data 1.
Antibodies	All ChIP-Seq antibodies are listed in the section "Antibodies" above and in Supplementary Table 1.
Peak calling parameters	Peaks of histone modifications were called using Macs2. H3.3/H3.3-G34W enrichment regions were called using a custom procedure. All necessary details are available in Methods.
Data quality	Quality was controlled using standard QC metrics in deepTools2.
Software	The raw sequence reads were processed using a standard pipeline (cutadapt,bowtie2). Read coverage was summarized and normalized using deepTools2. All details are available in Methods.
