# Supplementary material for: Globally altered epigenetic landscape and delayed osteogenic differentiation in H3.3-G34W-mutant giant cell tumor of bone
Source: Nat Commun. 2020 Oct 27;11:5414. doi: 10.1038/s41467-020-18955-y (PMC7591516; doi:10.1038/s41467-020-18955-y)
Supplement: Supplementary file 9 — Source Data [file 41467_2020_18955_MOESM9_ESM.zip › Source Data 2.pdf]

Supplementary Figure 1a

Original Western blot images

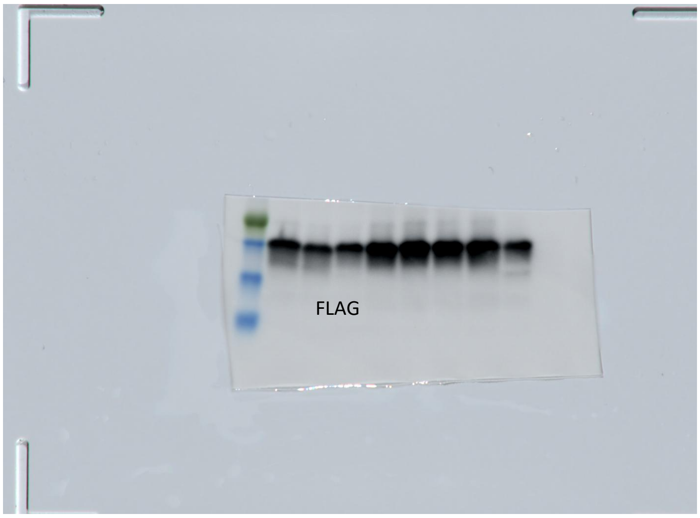

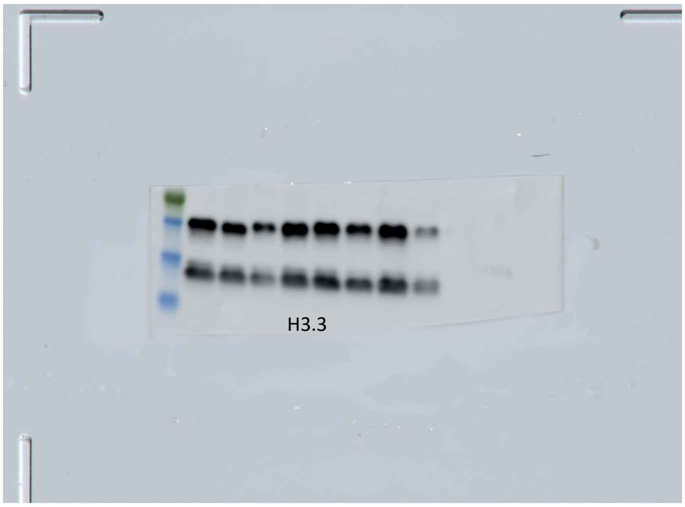

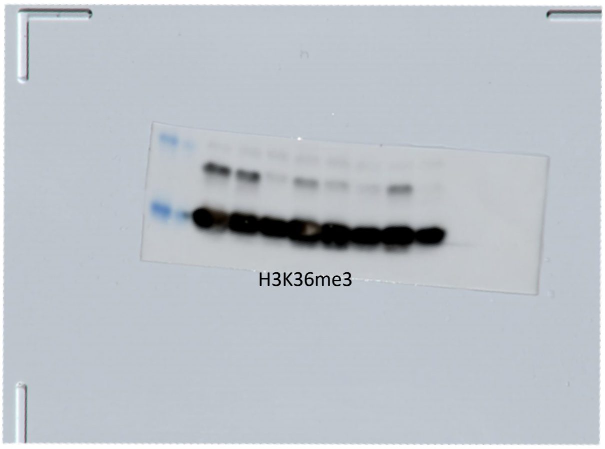

Supplementary Figure 1b

Original gel images

Wildtype allele specific primer

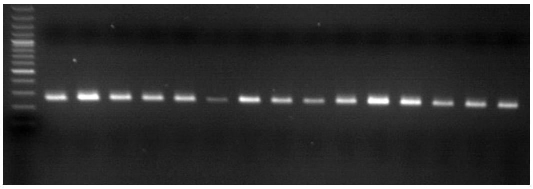

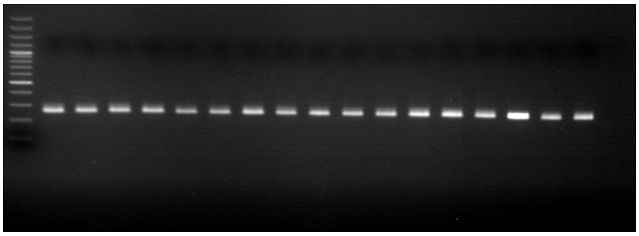

Mutant allele specific primer

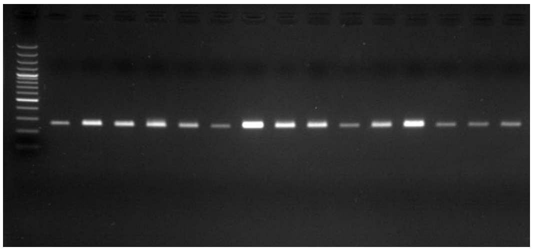

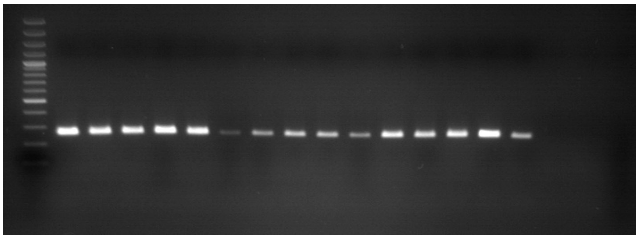

Supplementary Figure 2a

Unmodified, cropped

1 2 3 4 5 6 Lane

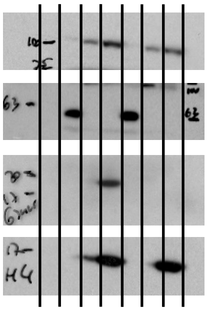

DAXX Marker line set closer to lane 1 than on the other blots

Tubulin

H3.3 G34W

H4

For this blot a non-chemiluminescence ladder was used, and the size marker was transferred manually to the film with ink.

Originals

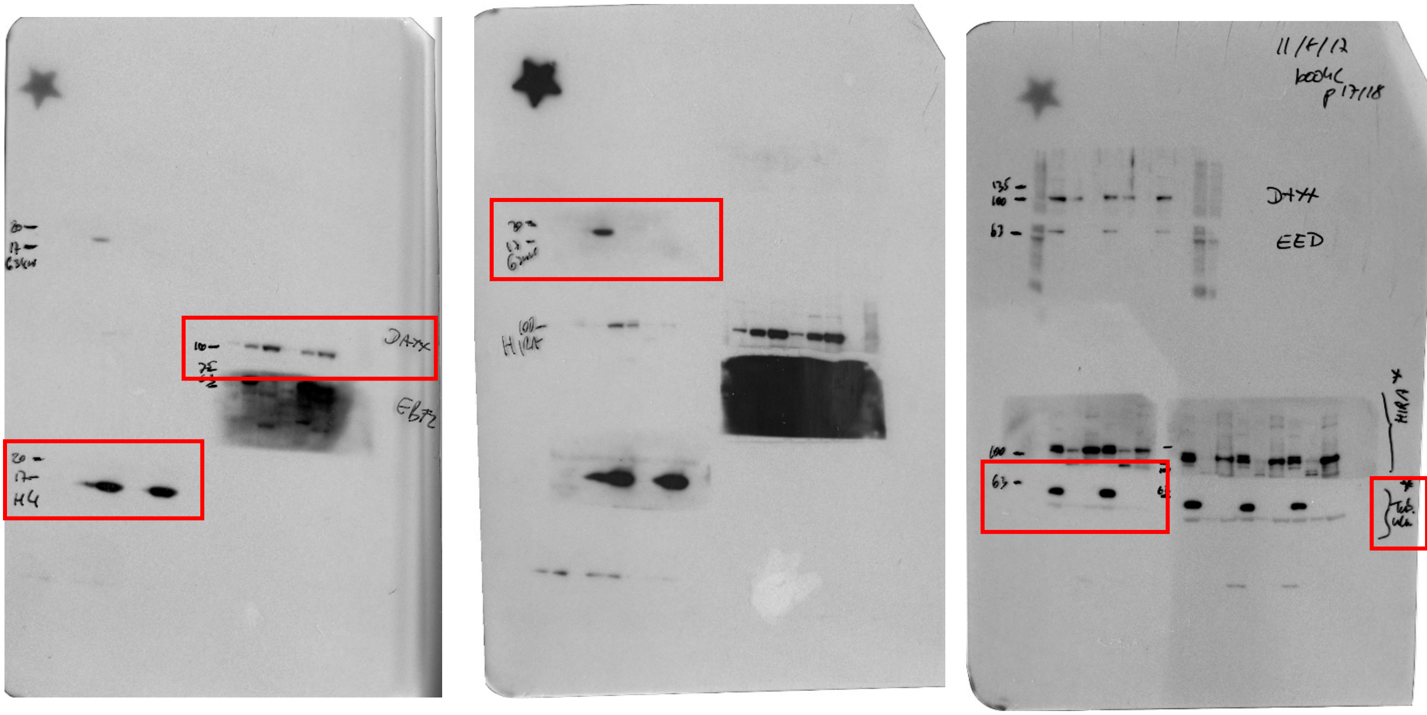

Supplementary Figure 2d

Originals

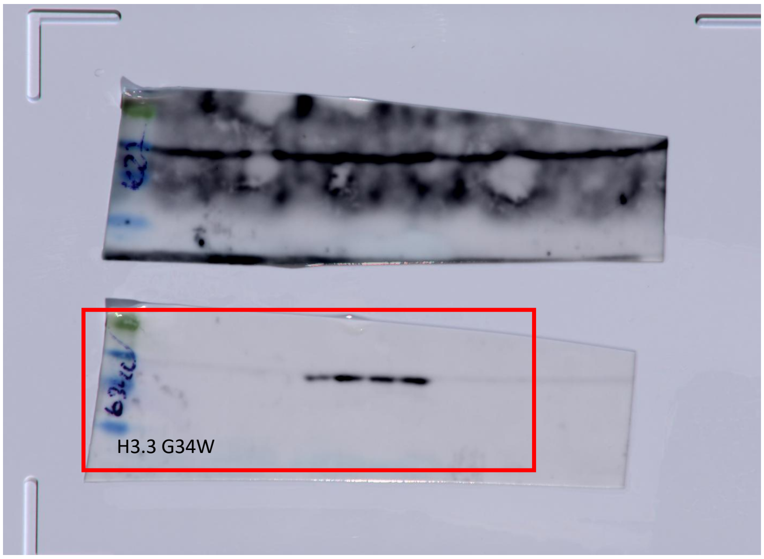

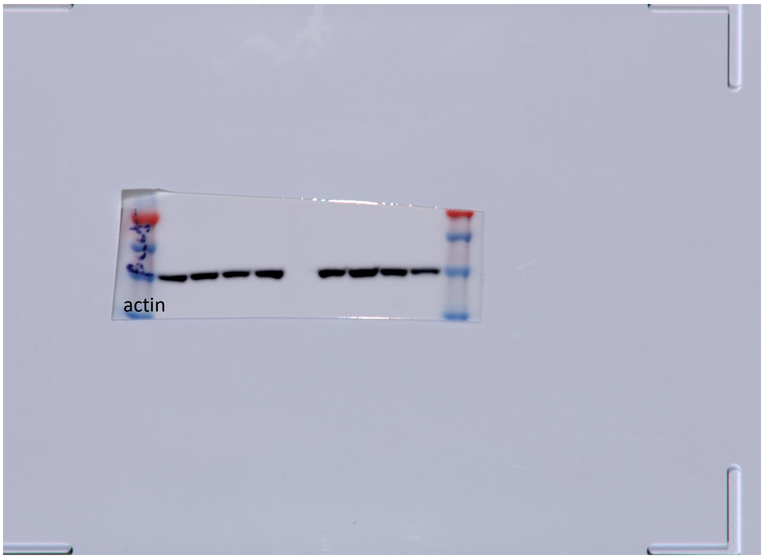

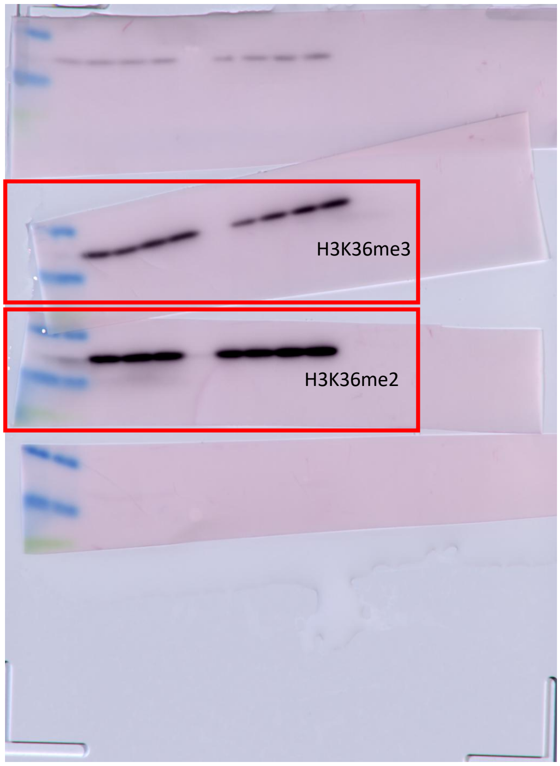

H3K36me2 lacks the first sample due to availability issues of the protein of this primary patient-derived cell line. Therefore the fist lane was not shown for H3.3 G34W, actin and H3K36me3 in the figure
